# Aggregation-induced emission of DNA fluorescence as a novel pan-marker of cell death, senescence and sepsis *in vitro* and* in vivo*

**DOI:** 10.7150/thno.122009

**Published:** 2026-01-01

**Authors:** Yiling Hu, Xiaoyan Wang, Ke Lu, Chonin Cheang, Yichen Liu, Yaru Zhu, Liming Xie, Moxin Li, Qingqing Zhou, Wenshan Yan, Ying Hou, Hongjie Zhang, Weiming Zheng, Changhuo Xu, Xiuping Chen, Han-Ming Shen, Zhen-Ju Huang, Yi-Hao Chen, Yi Zhou, Pei-Lin Cai, Hao Zhong, Lu Liu, Xi-Ping Wu, Xingxing Liu, Xiaolin Li, Ning Zeng, Yehuda G. Assaraf, Tzu-Ming Liu

**Affiliations:** 1 Institute of Translational Medicine, Faculty of Health Sciences & Ministry of Education Frontiers Science Center for Precision Oncology, University of Macau, Taipa, Macau, China.; 2 Centre of Reproduction, Development and Aging, Faculty of Health Sciences & Ministry of Education Frontiers Science Center for Precision Oncology, University of Macau, Taipa, Macau, China.; 3 State Key Laboratory of Quality Research in Chinese Medicine, Institute of Chinese Medical Sciences, University of Macau, Taipa, Macao, China.; 4 Department of Hepatobiliary Surgery I, General Surgery Center, Zhujiang Hospital, Southern Medical University, China.; 5 Guangdong Provincial Clinical and Engineering Center of Digital Medicine, Guangzhou 510280, China.; 6 Department of Critical Care Medicine, Zhujiang Hospital, Southern Medical University, Guangzhou 510280, China.; 7 Pulmonary and Critical Care Medicine, Zhujiang Hospital, Southern Medical University, Guangzhou 510280, China.; 8 Breast Cancer Center, Zhejiang Cancer Hospital, Hangzhou Institute of Medicine (HIM), Chinese Academy of Sciences, Hangzhou, Zhejiang 310022, China.; 9 The Fred Wyszkowski Cancer Research Laboratory, Faculty of Biology, The Technion-Israel Institute of Technology, Haifa 3200003, Israel.; 10 Translational Medicine R&D Center, Zhuhai UM Science and Technology Research Institute, Zhuhai 519000, China.

**Keywords:** aggregation-induced emission, autofluorescence, cell death, senescence, DNA

## Abstract

**Background and Aim:** Effective monitoring of cell death and senescence is critical across biology and medicine, yet a universal *in situ* fluorescent marker has remained elusive. This study aimed to identify and characterize a novel, endogenous optical signal arising from cellular stress and to evaluate its potential as a universal, label-free biomarker for visualizing cellular fate in diverse physiological and pathological contexts.

**Material and Methods:** Autofluorescence was characterized in human cell lines, *C. elegans*, a mouse sepsis model, and human plasma samples using two-photon and fluorescence lifetime imaging microscopy. The molecular origin of the signal was investigated through lysosome isolation, high-throughput DNA sequencing, gel electrophoresis, and photophysical experiments with synthetic nucleic acid oligonucleotides. The underlying routes to lysosomal accumulation were probed using shRNA-mediated knockdown and dominant-negative protein expression.

**Results:** We discovered that cytosolic DNA fragments, sequestered into lysosomes via ESCRT-mediated microautophagy, aggregate and exhibit strong aggregation-induced emission (AIE) with a distinct peak at ~600 nm. This signal originates from excimer formation in single-stranded DNA, with mitochondrial DNA being a primary source during cell death. This label-free fluorescence allowed for the differentiation of cell death types *in vitro* and its intensity correlated with the progression of aging in *C. elegans* and sepsis in both mice and a human pilot study.

**Conclusions:** Aggregated nucleic acids within lysosomes represent a novel, universal, and label-free endogenous biomarker for cell death and senescence. This AIE-based optical signal provides a powerful tool for real-time *in situ* visualization and dynamic tracking of cellular fate, holding significant potential for applications in basic research, diagnostics, and therapeutic monitoring.

## Introduction

Programmed cell death (PCD) is a genetically regulated process of cellular death execution that occurs as part of an organism's development, homeostasis, and response to environmental cues 1-3. It is a critical event in biology that eliminates unnecessary or severely damaged cells to facilitate embryogenesis, prevent collateral injury to nearby tissues, and coordinate immune responses 4. Over the past decades, various types of PCD have been identified 5, each characterized by distinct molecular mechanisms that give rise to unique cellular hallmarks and morphological phenotypes 6. Visualizing and assessing the occurrence, temporal dynamics, and spatial distribution of these cell death processes are essential for advancing studies in pathophysiological states, drug treatment responses, developmental biology, immune regulation, wound healing, and aging. Investigating PCD dynamics, as opposed to static assays, can provide novel molecular and mechanistic insights into biological processes, disease pathology, and pharmacodynamics. For instance, time-lapse assessments have revealed that ferroptosis in a localized group of cells can induce large-scale cell death by amplifying and spreading of ferroptotic waves 7. This discovery suggests that, similar to apoptosis, ferroptosis is involved in fast tissue clearance during embryonic development; Sublethal mitochondrial apoptotic stress, which can involve transient mitochondrial outer membrane permeabilization (MOMP), triggers the release of mitochondrial DNA (mtDNA) 8 that can drive cell senescence and age-related tissue dysfunction 9. Researchers concluded that sublethal mitochondrial apoptotic stress is a major driver of the senescence-associated secretory phenotype (SASP) 10; Dynamic studies of cell death revealed that a modest increase in the expression of senescence-associated protein p21 creates a “Goldilock zone” for proliferation, wherein cancer cells can survive chemotherapy 11,12. This counter-intuitive role for early p21 dynamics in cell-fate decisions pinpoints a source of proliferative cancer cells that can emerge following exposure to non-lethal doses of chemotherapy. These examples highlight the need for molecular imaging tools capable of visualizing the dynamics of cell death in various pathophysiological contexts. Currently however, there is a lack of *in vivo* reporter systems that can effectively capture and visualize various cell death dynamics *in situ*.

While fluorescent reporter systems exist for studying the temporal dynamics of apoptosis *in vivo* 13,14 and necroptosis *in vitro* 15, they often require genetic modification, which is time-consuming and may alter the biological environment. Therefore, there is a pressing need to develop a sensitive visualization method that can easily monitor the occurrence of PCD and track their fate and dynamics in real-time within cells without affecting their native state. In this respect, we recently identified a significant increase in apoptosis-specific red autofluorescence both *in vitro* and *in vivo* 16. Moreover, fluorescence lifetime imaging allowed us to further differentiate necrotic cells from apoptotic ones. These label-free hallmarks have been employed to evaluate the time-course pharmacodynamics in tumor organoids without relying on cell fixation 17. This discovery suggests that, by elucidating the origin and molecular mechanism of this endogenous autofluorophore, it could serve as a universal cell death marker for real-time monitoring of various PCD dynamics within their native biological environments, offering a powerful tool for researchers and possibly clinicians across multiple disciplines.

In this study, we confirmed that lysosomal-aggregated mitochondrial DNA (Ag-mtDNA) is responsible for the characteristic red autofluorescence observed in apoptotic cells. Under tightly focused excitation at 561 nm or two-photon near-infrared excitation at 1060 nm, the π-π stacking of deoxynucleotides in single-stranded DNA (ssDNA) fragments generate a distinct excimer autofluorescence at ~600 nm 18,19. Importantly, double-stranded DNA (dsDNA), including that present within nuclei, does not emit fluorescence under these excitation conditions. Since all forms of PCD involve certain types of mitochondrial pore formation 20-22, the translocation of cytosolic mtDNA into lysosomes further generates ssDNA fragments and activates the AIE in acidic environments. The types of PCD can be further differentiated by analyzing the intracellular distribution and fluorescence lifetime of Ag-mtDNA. Beyond the cell death indicator, we further show that the Ag-mtDNA fluorescence can also reflect cell senescence in aging organisms and the pathology dynamics of sepsis in an animal model and in a human pilot study. Our findings present a unique, label-free method to monitor cell death dynamics, offering a significant advancement over conventional static approaches that often require cell fixation, staining, and/or invasive reporters. This molecular imaging of the universal cell death marker Ag-mtDNA holds significant potential for studying cell death dynamics across a wide spectrum of biomedical disciplines.

## Materials and Methods

### Cell culture

Human breast cancer MDA-MB-231 cells (ATCC), L929 mouse fibroblasts (ATCC) and ATG7 knockout Mouse Embryonic Fibroblasts (MEF) cells (a generous gift from the laboratory of Prof. Han-Ming Shen at University of Macau) were grown in Dulbecco's Modified Eagle Medium (DMEM) (Gibco) supplemented with 10% fetal bovine serum (FBS), 1% penicillin-streptomycin (Gibco) and incubated at 37 °C in a 5% CO_2_ atmosphere. For experiments of cell viability and drug sensitivity, cells were seeded at a density of 5×10⁵ cells/mL in glass-bottom Petri dishes and allowed to reach 60-80% confluency before drug treatment was initiated.

### Drug treatment to induce various programmed cell death

Mouse fibroblast L929 cells were subjected to various treatments to induce different forms of cell death and mitophagy. For apoptosis, cells were treated with 30 μM cisplatin for 24 h. Ferroptosis was induced using 10 μM Erastin for 24 h, while pyroptosis was triggered by 5 mM Metformin hydrochloride for 24 h. Necroptosis was achieved with a combination treatment of 100 ng/mL tumor necrosis factor TNF-α, 20 μM caspase inhibitor Z-VAD-FMK, and 0.56 nM Smac mimetic SM-164 for 24 h. To inhibit necroptosis, cells were pre-treated with 40 μM Necrostatin-1 for 1 hour, followed by incubation with the TNF-α, Z-VAD-FMK, and SM-164 cocktail for 24 h. Mitophagy was induced using 50 μM carbonyl cyanide m-chlorophenyl hydrazine (CCCP) for 2 h. CCCP, a protonophore uncoupler is a well-established potent oxidative phosphorylation inhibitor.

### Multiphoton microscopy imaging and spectral analysis

For the imaging of cell red autofluorescence, cells were cultured in bottom-glass confocal dishes and maintained in a microincubator system compatible with the microscope, set to 37 °C with 5% CO_2_ (Nikon Instrument Inc., Japan). A tuneable near-infrared femtosecond laser, operating between 700 and 1300 nm (InSight X3, Spectra-Physics), was used as the excitation source for two-photon fluorescence microscopy imaging. Imaging was performed using a Nikon Eclipse Inverted Multiphoton Microscope (A1MP + Eclipse Ti-2E, Nikon Instrument Inc., Japan), equipped with a 40× water-immersion objective (NA = 1.15). The system collected both the excited red autofluorescence and second harmonic generation (SHG) signals through the same objective. These signals were then reflected by a multiphoton dichroic beam splitter and detected by four photomultiplier tubes (PMTs). Specifically, red autofluorescence was excited at 1060 nm and detected by the PMT in channel 3 (detection range: 604-679 nm). For wavelength-dependent excitation experiments, laser power after the objective was consistently adjusted to 15 mW across different excitation wavelengths. In each experimental group, three images were captured, each with a field of view of 317×317 μm, and mean fluorescence intensities were determined from at least 30 selected cells. Additionally, a CCD-cooled spectrometer (iDus 401 plus shamrock 193i, ANDOR, Oxford Instruments) was connected to the microscope's backside port. Following the acquisition of each multiphoton image, the corresponding two-photon emission spectra were recorded using the integrated spectrometer. This system also allowed for the detection of emission spectra from DNA samples, including oligonucleotides and mtDNA.

### Fluorescence lifetime data acquisition and analysis

To acquire fluorescence lifetime traces and perform fluorescence lifetime imaging microscopy (FLIM), we equipped the backside port of the same multiphoton microscope (Eclipse Ti-2E, Nikon) with two photon-counting photomultiplier tubes (PMTs) (PMC-150-4, Becker & Hickl), which shared the signal light path with the fluorescence spectrometer. Fluorescence lifetime measurements were recorded using a time-correlated single-photon counting (TCSPC) system (SPC-160, Becker & Hickl), synchronized with the scanning excitation of the Nikon A1 MP+ multiphoton microscope. Excited at 1060 nm, we measured the lifetime traces of two-photon red autofluorescence. To enhance the accuracy of the lifetime fitting, each FLIM image was acquired over 120 s, ensuring that the peak photon counts in the traces exceeded 200 in the majority of cell pixels. A pixel dwell time of 25.21 μs was used to generate FLIM images with 256 × 256 pixels. We used an instrument response function (IRF) convoluted two-component exponential decay model 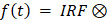
(a_1_e^-t/τ1^ + a_2_e^-t/τ2^) to fit the decay traces, where τ1 and τ2 represent short and long-lifetime components, and a_1_ and a_2_ are relative fraction of the lifetime components (a_1_ + a_2_ = 1).

### Lysosome isolation and DNA sequencing

A total of 200 mg of human breast cancer MDA-MB-231 cells were used for lysosome isolation. After the cells were detached by trypsinization, centrifuged at 1,100 rpm for 3 min, the supernatant was removed. The remaining cells weighing 200 mg were collected for the experiment. Cells were mechanically disrupted using a Dounce homogenizer in Lysosome Enrichment Reagent A (Thermo Fisher, 89839) until 80% lysis was achieved. Lysosome enrichment was then performed following the protocol provided in the Lysosome Enrichment Kit (ThermoFisher, 89839). The isolated lysosomes were lysed with 10% sodium dodecyl sulphate (SDS) for 10 min (50 mM Tris-HCl, pH 7.4). The resulting lysate was submitted to Beijing Youji Technology Co., Ltd (Haidian District, Beijing, China) for DNA sequencing and subsequent analysis.

### Western blotting and DNA electrophoresis

L929 cells were seeded at a density of 1.2×10⁶ cells/ well in six-well plates. After 24 h, cells were treated to induce apoptosis, pyroptosis, ferroptosis, necroptosis, and mitophagy. Following treatment, the supernatant was removed, and cells were washed twice with phosphate-buffered saline (PBS). Then, each well received 100 μL of cell lysis buffer (50 mM Tris, pH 7.4, 150 mM NaCl, 1% NP-40 detergent, 0.5% sodium deoxycholate, 0.1% SDS, with 1× phosphatase and protease inhibitor cocktail (ab271306) in H_2_O). The lysates were centrifuged at 14,000 × g for 5 min at 4 °C, and the supernatant was collected. Protein concentrations were determined using the Bio-Rad protein assay kit (reagent A: 500-0113; reagent B: 500-0114; reagent C: 500-0115). Equal amounts of protein (at least 15 μg per sample) were separated on Tris-glycine PAGE gels and transferred to 0.45 μm polyvinylidene fluoride (PVDF) membranes (Millipore) using the Trans-Blot SD semi-dry transfer system (Bio-Rad). Membranes were then blocked with PBS-Tween blocking buffer (5% milk powder, 0.05% Tween-20 in PBS) and incubated overnight at 4 °C with primary antibodies against β-actin (Abcam, diluted 1:1000), PINK1 (Cell Signaling Technology, diluted 1:1000), Parkin (Proteintech, diluted 1:1000). Secondary antibodies were applied for the detection of secreted proteins. Immunoblot signals were visualized using enhanced chemiluminescence (ECL) and recorded with Bio-Rad Chemidoc imaging system. The intensity of detected protein bands was quantified using ImageJ software. Protein expression levels were normalized by comparing the levels of secreted proteins to those in the corresponding cell lysates. For agarose gel electrophoresis, 100 ng of DNA was loaded onto a 0.5% agarose gel, and the gel was run at 85 Volt for 90 min. In parallel, 100 μM DNA oligonucleotides were loaded onto a 20% polyacrylamide gel and electrophoresed at 85 Volt for 90 min. After electrophoresis, the gels were imaged using the Bio-Rad Chemidoc imaging system.

### Cell death analysis by flow cytometry

Cell death induction was assessed using an Annexin V-FITC/ PI kit (Invitrogen). Following treatment, cells (5×10⁵) were collected, gently washed with PBS, and resuspended in 195 μL of binding buffer. To stain with Annexin V, 5 μL of Annexin V-FITC were added to the cell suspension and incubated for 15 min at room temperature in the dark. After incubation, cells were washed with binding buffer and resuspended in 200 μL of buffer containing 10 μL of propidium iodide (PI). Fluorescence signals were measured using a BD Accuri™ C6 Cytometer (BD Biosciences, USA) with FITC and PI detection channels, and data were analyzed with FlowJo software (Tree Star). Forward scatter (FSC) and side scatter (SSC) signals were employed for gating, and a minimum of 10^4^ cells were analyzed per sample. Single-label controls for PI and FITC were used to adjust for calibrating background fluorescence. Thresholds were set to define positive and negative cell populations for Annexin V and PI staining. Cells were classified as healthy if both Annexin V and PI were negative, apoptotic if Annexin V was positive and PI was negative, and necrotic or late apoptotic if both Annexin V and PI were positive.

### Cell death analysis by real-time PCR

L929 cells were seeded at a density of 4×10^5^ cells/well in six-well plates. After inducing the desired conditions, total RNA was extracted from the cells using the Tiangen RNA extraction kit (DP451) according to manufacturer's protocol. cDNA synthesis was performed using the iScript™ cDNA Synthesis Kit (Bio-Rad). The cDNA was then subjected to quantitative real-time PCR using iTaq Universal SYBR Green Supermix. The following primers were used for PCR amplification, Hox1: Forward: 5'-TGAAGGAGGCCACCAAGGAGG-3', Reverse: 5'-AGAGGTCACCCAGGTAGCGG-3', Acsl4: Forward: 5'-GGGCTGGATCTTATGGTGGT-3', Reverse: 5'-CCACCCACACCATCTCCTTA-3', RIPK1: Forward: 5'-GAGAGCGTCCTTGTGGGACT-3', Reverse: 5'-CCGGTGAAGTTGGTCGTAGA-3', RIPK3: Forward: 5'-CTGGATGTGGAGTGGAGGTC-3', Reverse: 5'-TTGGCTTCAGGGAGAGTGTG-3', IL-18: Forward: 5'-GACTCTTGCGTCAACTTCAAGG-3', Reverse: 5'-CAGGCTGTCTTTTGTCAACGA-3', IL-1β: Forward: 5'-GCAACTGTTCCTGAACTCAACT-3', Reverse: 5'-ATCTTTTGGGGTCCGTCAACT-3'.

### shRNA lentivirus production and infection

For the shRNA knockdown experiment, lentiviral constructs were employed, starting with sequence design and vector construction. The sequences for SLC3A2 were designed as follows: SLC3A2-F1: 5'-ccggGGTGGAGCTGAATGAGTTActcgagTAACTCATTCAGCTCCACCtttttg-3', SLC3A2-R1: 5'-aattcaaaaaGGTGGAGCTGAATGAGTTActcgagTAACTCATTCAGCTCCACC-3'; SLC3A2-F2: 5'-ccggGCCTACTCGAATCCAACAActcgagTTGTTGGATTCGAGTAGGCtttttg-3', SLC3A2-R2: 5'-aattcaaaaaGCCTACTCGAATCCAACAActcgagTTGTTGGATTCGAGTAGGC-3'. The pLKO.1-TRC vector was used for this purpose. The digestion protocol involved treating the pLKO.1 TRC-cloning vector with AgeI, using 6 μg of the vector, 5 μL of 10× NEB buffer 1, and 1 μL of AgeI, with ddH_2_O added to bring the total volume to 50 μL. The mixture was incubated at 37 °C for 2 h. Thereafter, the eluate was digested with EcoRI in a reaction containing 30 μL of the AgeI-digested pLKO.1 TRC-cloning vector, 5 μL of 10×NEB buffer for EcoRI, 1 μL of EcoRI, and 14 μL of ddH_2_O, and the incubation was repeated at 37 °C for 2 h. The digested DNA was then resolved on a 0.8% agarose gel for 45 min. The 7 kb band was excised and purified using the Qiaquick gel extraction kit, and the DNA was eluted in 30 μL of ddH_2_O. For shRNA sequence annealing, 5 μL of the forward oligonucleotide, 5 μL of the reverse oligonucleotide, 5 μL of 10×NEB buffer, and 35 μL of ddH_2_O were combined. The mixture was heated at 95 °C for 4 min, incubated at 70 °C for 10 min, and then allowed to slowly cool to room temperature over several hours. For the ligation step, 2 μL of the annealed oligonucleotide, 20 ng of the digested pLKO.1 TRC-cloning vector, 2 μL of 10×NEB T4 DNA ligase buffer, and 1 μL of NEB T4 DNA ligase were combined, with ddH_2_O added to bring the total volume to 20 μL. The ligation reaction was then incubated at 16 °C overnight. For the lentiviral transfection and packaging process, 1 μg of the pLKO.1 shRNA plasmid, 750 ng of the psPAX2 packaging plasmid, and 250 ng of the pMD2.G envelope plasmid were transfected into HEK-293T cells using Lipo3000. The cell culture supernatants containing the lentivirus were collected at 48 and 72 h post-transfection. For the lentiviral infection, the viral supernatant was added to 231 cells in the presence of 8 μg/mL polybrene. After 24 h, the medium was replaced with fresh culture medium containing 4 μg/mL puromycin for selection. The knockdown efficiency was assessed using real-time PCR with the following primers: SLC3A2 Forward 5'-GACTGTGAAGGGCCAGAGTGAA-3' and SLC3A2 Reverse 5'-TGGTCCCAGTGGCGGATA-3'.

### Senescence cell model establishment and identification

L929 cells were seeded at a density of 4x10^5^ cells/well in a 6-well plate and cultured until they reached approximately 80% confluency. At this stage, the cells were exposed to 20 Gy of X-ray radiation for a total duration of 16 min and 18 s to induce cellular senescence. Following irradiation, the cells were incubated for an additional 72 h to allow the senescence process to develop. After the incubation period, the Senescence Cell Detection Kit (Beyotime, C0602) was used according to the manufacturer's protocol to assess β-galactosidase activity, which serves as a marker for cellular senescence.

### Mitochondrial DNA extraction

MDA-MB-231 cells (5 × 10^6^) were harvested by centrifugation at 600 × g for 5 min. The cell pellet was then washed with 5-10 mL of ice-cold PBS, followed by centrifugation at 600 × g for 5 min at 4 °C. After carefully discarding the supernatant, mtDNA extraction was performed using the Mitochondrial DNA Isolation Kit (Abcam, ab65321), following the manufacturer's protocol.

### Cecal ligation and puncture (CLP) mouse model

All animal experiments were approved by the Animal Facility of the Faculty of Health Sciences at the University of Macau (Approval number UMARE-027-2021). Male C57BL/6 mice (8-12 weeks old, 20-25 g) were obtained from the Laboratory Animal Center of the Faculty of Health Sciences, University of Macau. All animals were housed in a specific pathogen-free (SPF) facility under controlled temperature conditions and maintained on a 12-hour light/dark cycle. All experimental procedures adhered to the ethical guidelines for animal research set by the University of Macau. To establish CLP model of sepsis, mice were anesthetized with an intraperitoneal injection of 300 µL of 4% Avertin (Sigma-Aldrich). The shaved abdominal area was disinfected with 75% ethanol, a midline skin incision (1-1.5 cm) was made, and the cecum was gently exposed and ligated at approximately 60-70% of its length. The cecum was then punctured twice with a 23-gauge needle. After repositioning the cecum, the incision was closed with 5-0 silk sutures. Mice in the sham-operated group underwent the same surgical procedure but without cecal ligation and puncture. Following surgery, all mice received intraperitoneal injections of 1 mL sterile saline. Blood Samples were collected at 8,12,16 and 24 h post-surgery. Blood samples were centrifuged at 3000 rpm for 10 min at 4 °C, and plasma was collected for further analysis.

### Imaging of *Caenorhabditis elegans*

The *C. elegans* strains were obtained from *Caenorhabditis* Genetics Center (CGC) and maintained on nematode growth medium plates, seeded with OP50 *E.coli*, as previously described 23. The temperature-sensitive CB1370 *daf-2(e1370)III* mutants were grown at 16 °C. The wild-type Bristol strain N2 and CF1038 *daf-16(mu86)I* mutants 24 were grown at 20 °C. All worms were passed down for more than three generations without contamination and starvation. For two-photon imaging, live worms at different stages were anesthetized in 5 μL of 10 mM sodium azide (Sigma-Aldrich, S2002) on glass slides and observed directly.

### Clinical study

Our sepsis clinical study employed a case-control design to enroll participants from two groups: a healthy control group (n = 22) and a sepsis group of patients (n = 14) at Zhujiang Hospital in Guangzhou, China, between August 2023 and October 2024. The healthy control group consists of physically healthy individuals with no underlying medical conditions and no history of hospitalization in the past two years. In contrast, the sepsis group includes patients with suspected infections who meet the criteria for a Sequential Organ Failure Assessment (SOFA) score ≥ 2, as confirmed by a physician in accordance with the Sepsis-3 definition 25. Exclusion criteria include minors, pregnant individuals, individuals with mental illnesses, and other vulnerable populations.

Each participant provided a 10 mL blood sample for the measurement of procalcitonin (PCT) levels and blood autofluorescence intensity. Venous blood samples from the sepsis group were collected within 24 h of hospital admission. This study did not involve any therapeutic interventions, and fluorescence intensity measurements did not influence clinical diagnosis or treatment. Blood samples were collected using heparin as an anticoagulant. Plasma was separated by centrifugation at 1500 × g for 15 min at 4 °C and stored at -80 °C to prevent oxidation of fluorescent metabolites. The fluorescence spectra of all plasma samples were measured within three weeks of collection. This human clinical study has been ethically approved by the Institutional Review Board of Zhujiang Hospital (Approval No. 2022-KY133-01), and all participants have signed informed consent forms.

### Statistical analysis of the intensity and lifetime of cell red autofluorescence

**Figure 1:**
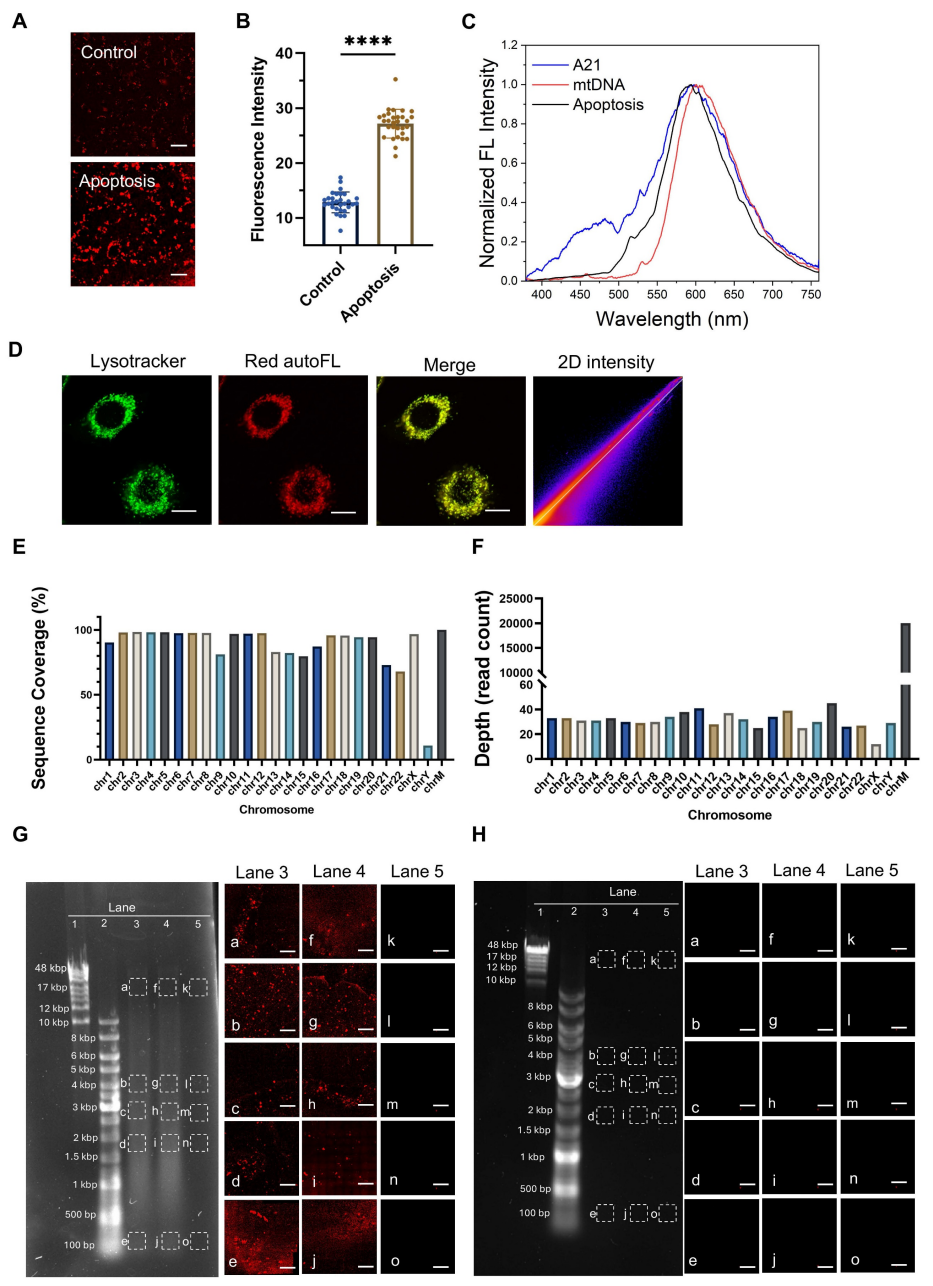
**Characteristics of red autofluorescence in apoptotic cells and NGS analysis of DNA present in lysosomes.** (**A** and** B**) Two-photon fluorescence microscopy (λ_ex_ = 1060 nm) and quantification of intracellular fluorescence intensities in human breast cancer MDA-MB-231 cells, with or without 30 μM cisplatin treatment (n ≥ 30). Scale bars: 50 μm, error bars represent the mean ± SD, ****: *p <* 0.0001. (**C**) Two-photon fluorescence emission spectra (λ_ex_ = 1060 nm) of red autofluorescence in MDA-MB-231 cells treated with 30 μM cisplatin for 24 h (black curve), poly A oligonucleotides (blue curve), and mtDNA in mitochondria (red curve). (**D**) Colocalization of lysosomes (λ_ex_ = 488 nm) with red autofluorescence signals (λ_ex_ = 561 nm) in cells exposed to 30 μM cisplatin for 24 h. Lysosomes were viably stained with LysoTracker Green. Scale bars: 10 μm. (**E** and** F**) High-throughput sequencing results showing genome coverage and sequencing depth after alignment with the complete cellular genome. ChrM denotes mitochondrial DNA. Agarose gel electrophoresis analysis of DNA extracted from lysosomes of (**G**) apoptotic and (**H**) control cells, with two-photon red fluorescence images a-o obtained by exciting 1-mm thick gel bands (λ_ex_ = 1060 nm) sliced from the regions indicated by dashed squares. Lane 1: 10kbp-48kbp ladder, Lane 2: 100bp-10kbp ladder, Lane 3: extracted lysosomal products from cells, Lane 4: further release of the DNA fragments by lysing the extracted lysosomal products with 10% SDS, and Lane 5: blank control.

**Figure 2:**
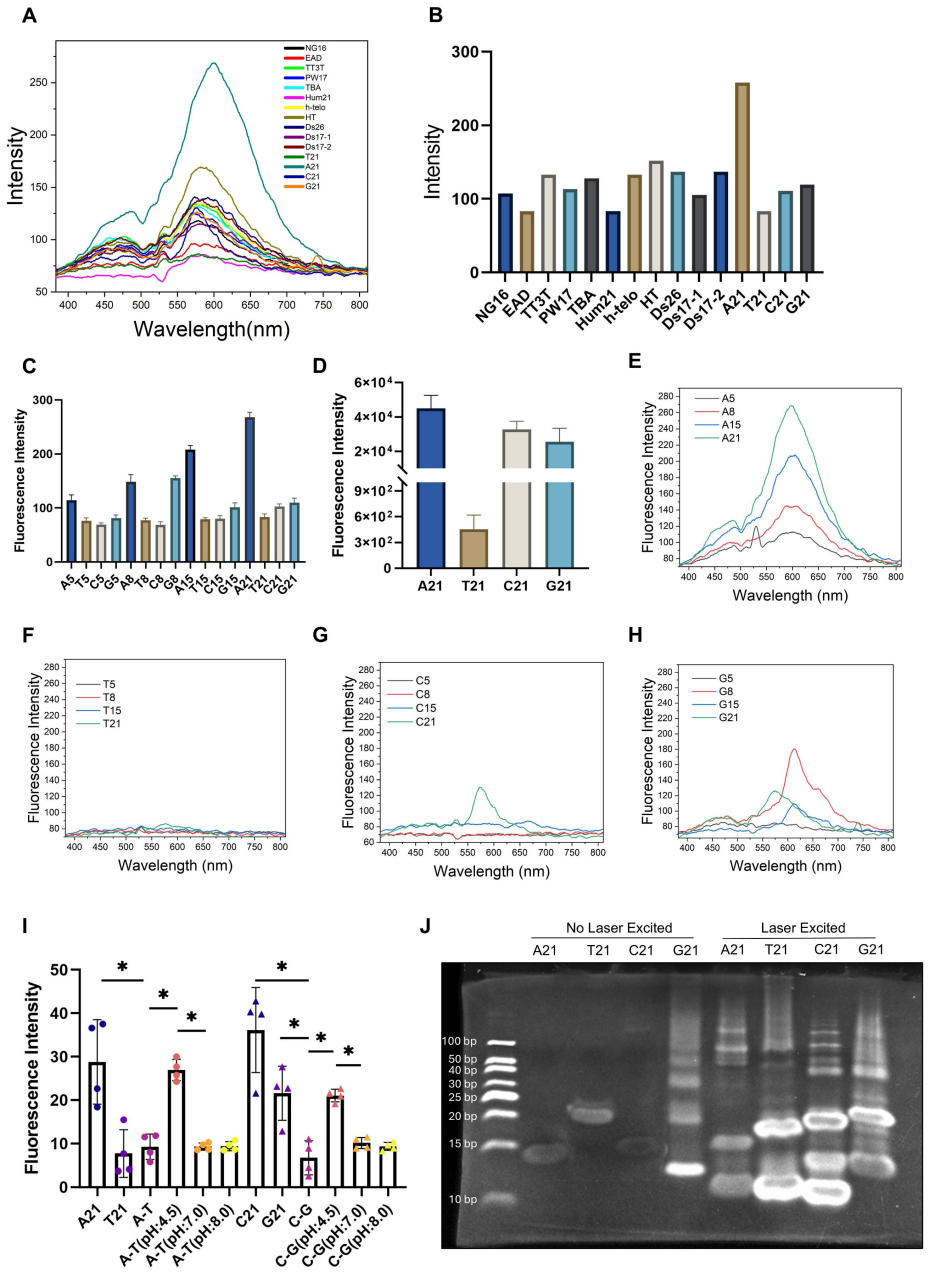
** Molecular mechanism of aggregation-induced emission (AIE) autofluorescence in DNA oligonucleotides. (A)** Two-photon fluorescence emission spectra (λ_ex_ = 1060 nm) and** (B)** fluorescence emission intensity of DNA oligos with various sequences. Two-photon fluorescence intensities (λ_ex_ = 1060 nm) of DNA nucleotide standards in (**C**) solution phase and (**D**) powder form. (**E** to **H**) Two-photon emission spectra of DNA oligonucleotides composed of A, T, C, and G nucleotides with lengths of 5, 8, 15, and 21 bases at a concentration of 100 μM in a 30 μL aqueous solution. (**I**) Two-photon fluorescence intensity analysis of 21-base single-stranded A, T, C, G oligonucleotides, their annealed A-T and C-G double-stranded forms, and the double-stranded forms adjusted to pH 4.5, 7.0 and 8.0 using hydrochloric acid and Sodium hydroxide. (n = 4) *: *p <* 0.05, error bars represent the mean ± SD. (**J**) PAGE images of 21-base A, T, C, G oligonucleotides before and after 1060 nm two-photon laser excitation.

**Figure 3:**
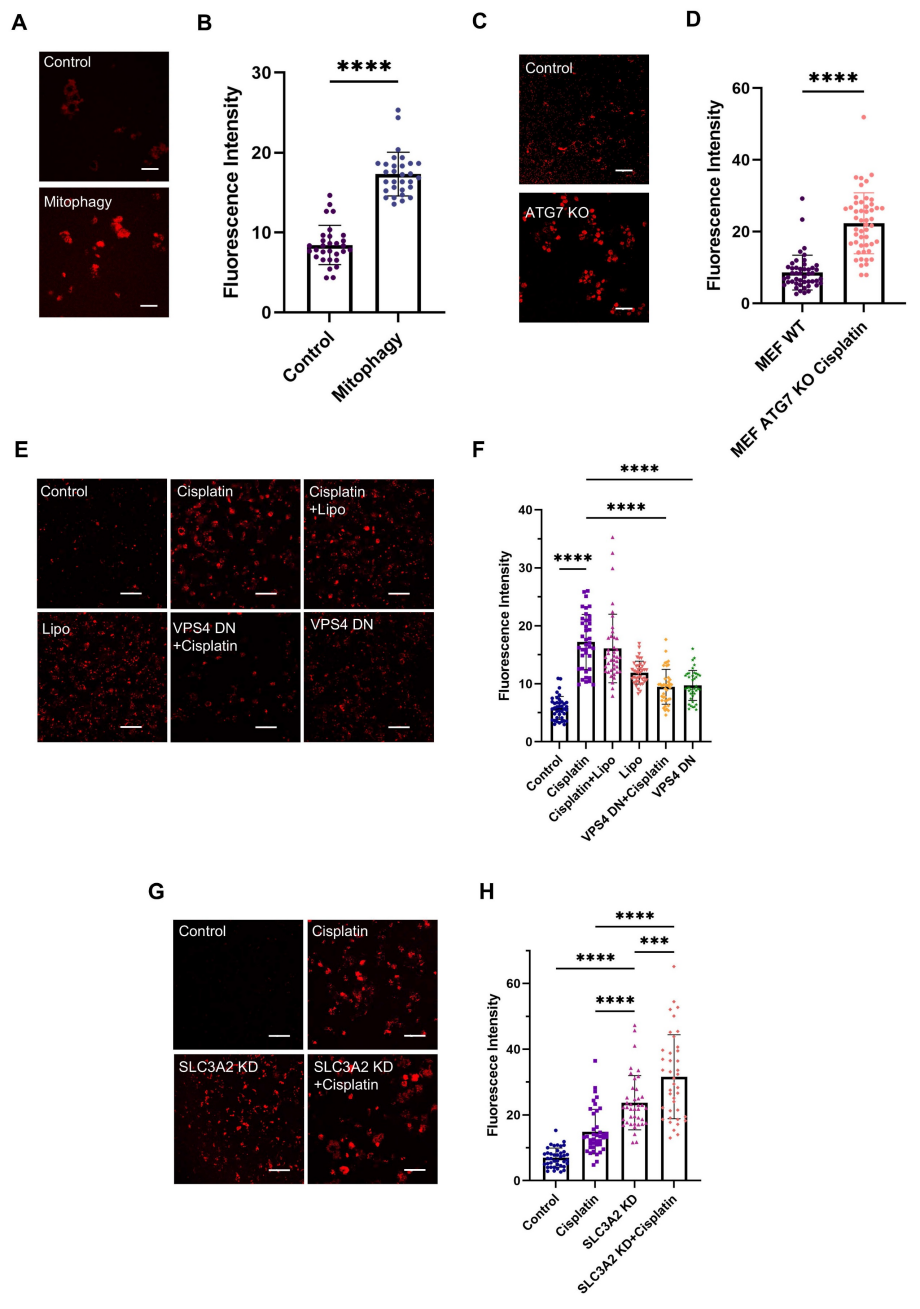
** Molecular mechanism of mtDNA translocation to lysosomes.** Two-photon red fluorescence images (λ_ex_ = 1060 nm) and intensity of (**A** and **B**) L929 cells (n = 30) undergoing mitophagy after 2-hour of treatment with 50 μM CCCP, Scale bars: 50 μm. (**C** and **D**) ATG7 knockout MEF cells (n = 50) after cisplatin-induced apoptosis, Scale bars: 50 μm. (**E** to **F**) MDA-MB-231 cells (n = 40) expressing a dominant-negative VPS4 mutant, treated with 30 μM cisplatin for 24 h, and (**G** to** H**) MDA-MB-231 cells (n = 40) with SLC3A2 knockdown via shRNA, treated with 30 μM cisplatin for 24 h. Scale bars: 50 μm, error bars represent the mean ± SD, ***: *p <* 0.001, ****: *p <* 0.0001.

**Figure 4:**
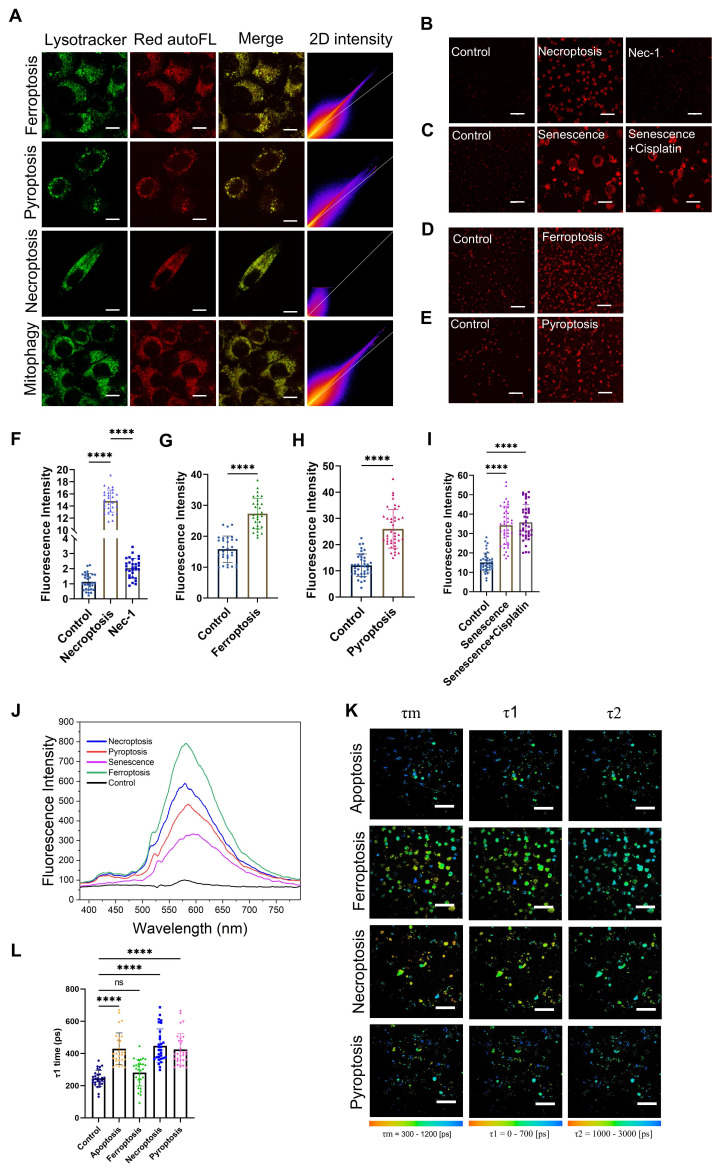
** Comparative analysis of Ag-mtDNA autofluorescence in ferroptosis, necroptosis, pyroptosis, and senescence.** (**A**) The characteristics of two-photon excited (λ_ex_ = 1060 nm) Ag-mtDNA autofluorescence (red) and lysosomal colocalization (Merge column) in cells undergoing ferroptosis, pyroptosis, necroptosis, and mitophagy. Lysosomes were stained with LysoTracker Green (λ_ex_ = 488 nm). The 2D intensity colocalization histograms were generated using ImageJ coloc2, Scale bars: 10 μm. (**B** to** I**) Two-photon (λ_ex_ = 1060 nm) red fluorescence images of L929 cells and corresponding intensity statistics undergoing (**B** and **F**) necroptosis and Necrostatin-1 (Nec-1) blockage (n = 30), (**C** and **I**) senescence (n = 40), (**D** and **G**) ferroptosis (n = 30), and (**E** and **H**) pyroptosis (n = 40). (**J**) Emission spectra of two-photon (λ_ex_ = 1060 nm) red fluorescence in cells undergoing ferroptosis, necroptosis, pyroptosis, and senescence. (**K** and **L**) Fluorescence lifetime imaging microscopy (λ_ex_ = 1060 nm) and statistical analysis cells during apoptosis, ferroptosis, pyroptosis and necroptosis. Scale bars: 50 μm, (n = 30) ns: not significant, error bars represent the mean ± SD, ****: *p <* 0.0001.

**Figure 5:**
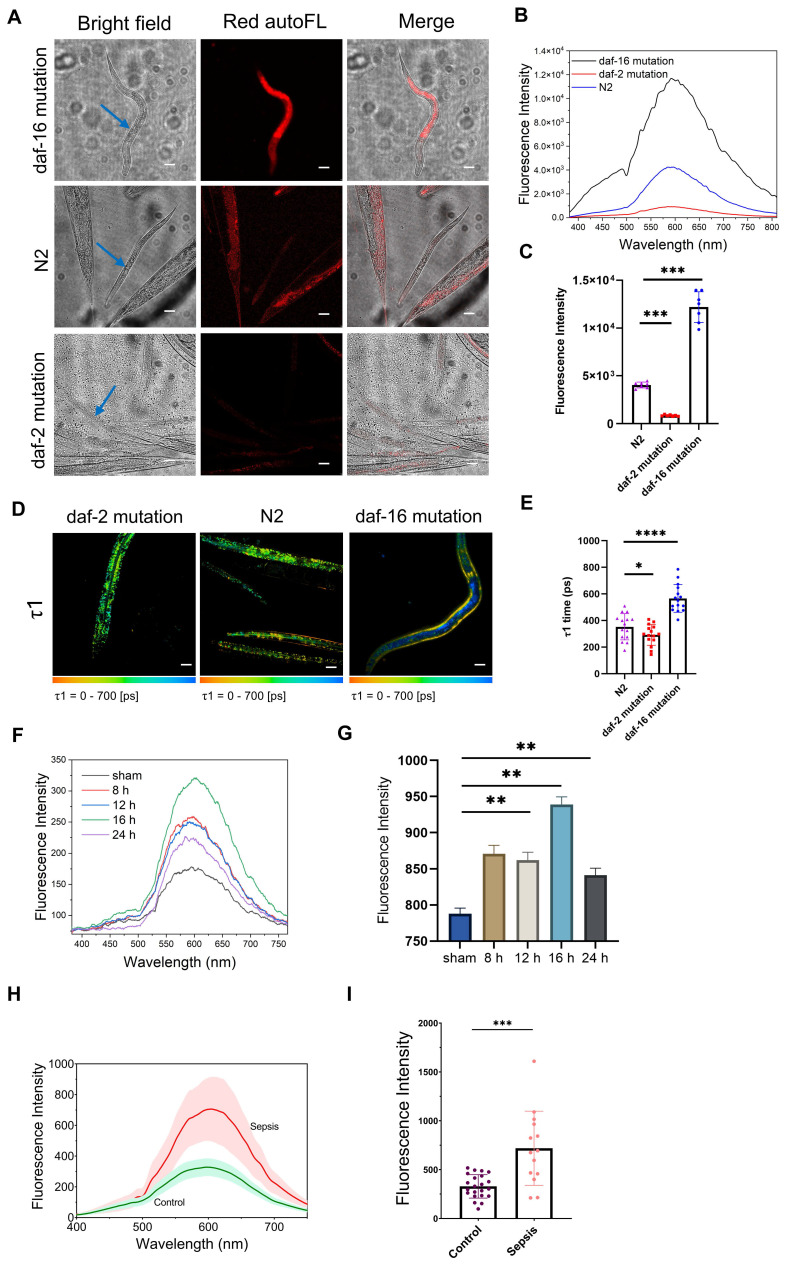
** Application of aggregated-DNA fluorescence in reporting aging in *C. elegans* and sepsis in mice and human.** (**A**) Bright field, two-photon (λ_ex_ = 1060 nm) red autofluorescence (autoFL) images and (**B**) spectra of promoting aged (daf-2 mutation), delaying aging (daf-2 mutation) and wild type (N2) *C. elegans*. Blue arrows indicate worms being measured with two-photon fluorescence spectra. Scale bars: 50 μm. (**C**) Fluorescence intensities of three *C. elegans* strains mentioned above (n = 6 for each strain). (**D**) fluorescence lifetime imaging microscopy of red autofluorescence in three *C. elegans* strains mentioned above, and (**E**) fitting parameters τ1 (n = 15). Scale bars: 50 μm. (**F**) Emission spectra of serum from septic mice under 1060 nm two-photon laser excitation. (**G**) The two-photon red autofluorescence intensities of septic mice serum at 600 nm (n = 6). (**H**) The average fluorescence emission spectra of plasma samples from healthy individuals (n = 22) and sepsis patients (n = 14) under 1060 nm two-photon excitation. The solid lines represent the mean fluorescence intensity of the control group (green) and the sepsis group of patients (red), while the shaded regions denote the standard error of the mean (SEM). At 600 nm, the fluorescence intensity of the sepsis group was markedly higher compared to the control group, indicating a significant difference in autofluorescence properties between the two groups. (**I**) Spectra of control and sepsis group. Fluorescence intensities of blood samples from healthy individuals (n = 22) and sepsis patients (n = 14). Mann-Whitney test, error bars represent the mean ± SD, *: *p <* 0.05, **: *p <* 0.01, ***: *p <* 0.001, ****: *p <* 0.0001.

The mean fluorescence intensity within individual cells was quantified using Fiji (ImageJ) software. Three images were captured per group, with a minimum of 30 cells analyzed for each condition. Fluorescence intensity within each cell was measured by calculating the mean fluorescence value. For fluorescence lifetime analysis, the FLIM data were processed with SPC Image software (Becker & Hickl). Statistical evaluation of the exported data was performed using unpaired Student's t-test (Figure 1B, Figure 3B and D, Figure 4G and H, Figure 5I, Figure S1B), Mann Witney U-test (Figure 2I, Figure 5C, E, G and I, Figure S5B-H, Figure S9B and D), One-way ANOVA with Tukey's post hoc analysis (Figure 3F and H, Figure 4F, I and L, Figure S7B), or Kruskal-Wallis test with Dunn's post hoc analysis (Figure S6D and E, Figure S9E) through GraphPad Prism (version 9.0). In the calculation of pH value from fluorescence intensity of the pH sensing dye, we used Mean ± 1.96 × SEM to calculate the 95% confidence interval, where SEM represents the standard error of mean in a sample. Emission spectrum graphs were generated using Origin software (version 8.6). Next-generation sequencing data were analyzed by Beijing youji technology Company.

## Results

### Apoptosis-specific autofluorescence originates from the aggregated mtDNA

In our previous studies 16,17, we employed the chemotherapeutic drug cisplatin, a gold-standard apoptosis inducer 26, to trigger programmed cell death in human MDA-MB-231 breast cancer cells and observed a significant increase in red autofluorescence intensity (Figure 1A-B). Cisplatin exerts its cytotoxic effect primarily through the generation of both interstrand and intrastrand DNA crosslinks. Subsequent DNA damage recognition initiates a coordinated activation cascade within cellular surveillance systems, engaging critical molecular regulators including the ATR kinase complex, p53/p73 tumor suppressor axis, and MAPK signaling networks. These interconnected pathways collectively drive the execution phase of programmed cell death mechanisms 26,27. Time- and dose-dependent studies on the observed red autofluorescence demonstrated a clear phenotype of increasing red autofluorescence with both higher cisplatin concentrations and longer incubation times 16. Two-photon fluorescence microscopy excitation at 1060 nm (Figure 1A) produces stronger emission signals compared to single-photon excitation at 561 nm (Figure S1A-B). The corresponding two-photon emission spectrum peaked at ~600 nm (Figure 1C, black curve). To comprehensively characterize this endogenous signal, we investigated the photophysical properties of aggregated polyadenine (polyA) oligonucleotides as a model system. The aggregates display broad absorption and fluorescence excitation spectra, with a prominent excitation range between 450-600 nm (Figure S2B-C), which is consistent with our use of 561 nm single-photon excitation. Correspondingly, single-photon excitation at 530 nm also generated the characteristic ~600 nm emission peak (Figure S2D). We determined the fluorescence quantum yield to be 0.14%, calculated using Rhodamine B as a reference standard (Table S1). While this quantum yield is modest, the high local concentration of aggregated DNA within lysosomes enables strong and reliable signal detection. Crucially, the signal also exhibited robust photostability, retaining substantial intensity even after 9 min of continuous two-photon laser irradiation, making it highly suitable for time-lapse imaging (Figure S2A). Using viable LysoTracker Green staining and colocalization analysis, we confirmed that this red autofluorescence in apoptotic cells was localized in lysosomes (Figure 1D and Table 1). It has been reported that dried-out condensed DNA oligonucleotides have a red autofluorescence around 600 nm 28,29. Using hydrated polyA oligonucleotides, we observed aggregated granules (Figure S1C) under fluorescence imaging and confirmed a similar emission spectrum (Figure 1C, blue curve) to apoptotic cells. Considering the spectral similarity and the mtDNA efflux in apoptosis 8, we hypothesized that the red autofluorescence signal in apoptotic cells may originate from mtDNA fragments. To corroborate this, we isolate lysosomes from apoptotic cells and subsequently extracted their DNA for high-throughput sequencing and DNA gel electrophoresis experiments. Sequencing coverage analysis revealed that lysosomes contain both nuclear and mitochondrial DNA (Figure 1E). However, sequencing depth analysis indicated that most of the DNA present in lysosomes was mtDNA (Figure 1F). We then we performed agarose gel electrophoresis on isolated lysosomes and their lysates. This technique is well established for resolving and identifying DNA fragments, and thus was employed to assess the presence of DNA in our samples. Following electrophoresis, we used two-photon excitation at 1060 nm to image the gel slices, enabling the visualization of red fluorescence associated with DNA aggregation. Notably, lysosomes isolated from apoptotic cells displayed distinct red, spot-like aggregates within the gel lanes (lanes 3 and 4 in Figure 1G). The absence of typical DNA fragment migration bands in the gel electrophoresis may be attributed to the geometric and rheological differences between aggregated DNA and relaxed double-stranded DNA. In contrast, no red fluorescent spots were observed in gels containing lysosomes from control cells (Figure 1H), and the gel itself exhibited no background fluorescence (lane 5 in Figure 1G). Furthermore, we found that the enriched and extracted mtDNA exhibited a 600-nm emission (Figure 1C, red curve) similar to that observed in apoptotic cells. To confirm that mtDNA enrichment in lysosomes is a general feature, we performed DNA sequencing on lysosomal fractions from cells undergoing necroptosis, pyroptosis, ferroptosis and senescence. In necroptosis, pyroptosis and ferroptosis, mtDNA was significantly more abundant than nuclear DNA (nDNA), confirming that mtDNA translocation to the lysosome is a conserved step in these cell death pathways. In senescent cells, both mtDNA and nDNA were found at comparable levels, suggesting a broader lysosomal turnover of nucleic acids in this state (Figure S3A-L).

### Denaturation and fragmentation of DNA are required conditions for aggregation-induced emission (AIE)

To examine the autofluorescence of DNA oligonucleotides, we used 1060 nm femtosecond pulses to excite single stranded oligo sequences composed of different bases (Table S2 and S3) and detected their emission spectra. We found a characteristic 600 nm two-photon emission peak (Figure 2A-B). Furthermore, to investigate the required condition for strong aggregation-induced emission (AIE), we prepared ssDNA oligonucleotides of different lengths to mimic denatured DNA fragments. In contrast to conclusions in previous literature 18, we found that these hydrated oligonucleotides and their solid powder form exhibited varying two-photon autofluorescence properties (Figure 2C-H, Figure S1D-E). Polyadenine oligonucleotide (A21) displayed the strongest autofluorescence at 600 nm, whereas polythymine oligonucleotide of the same length (T21) had barely detectable autofluorescence. These results are in accord with the ensemble fitting conclusions from a small-angle X-ray scattering investigation 19, where polyA oligonucleotides were found to form base stacking, whereas polyT did not. As the length of the polyA oligonucleotides decreased, their autofluorescence intensity gradually decreased (Figure 2C and E-H). In contrast, the autofluorescence of the other three bases oligonucleotides was relatively weaker and, in some cases, shifted away from 600 nm (Figure 2G-H).

Considering the structural differences between ssDNA and dsDNA, we annealed A21 with T21 and C21 with G21 to form A-T and C-G dsDNA, respectively. The results showed that the autofluorescence intensity of the annealed dsDNA was significantly lower compared to that of the single-stranded oligonucleotides (Figure 2I and Figure S1F). To mimic the denaturation of DNA induced in the acidic environment of lysosomes, we further adjusted the pH of the annealed dsDNA to 4.5 using hydrochloric acid. Interestingly, the denatured ssDNA regained its characteristic autofluorescence. In contrast, control samples maintained at neutral (pH 7.0) or alkaline (pH 8.0) conditions exhibited negligible fluorescence. (Figure 2I and Figure S1F). This finding validates that acidic denaturation and ssDNA aggregation are two necessary and sufficient conditions to yield activation of Ag-mtDNA fluorescence.

We further evaluated the effect of light excitation on the aggregation of DNA by exciting femtosecond laser excitation of oligonucleotide samples for 1 min, whereas control samples were left unexcited. We then performed polyacrylamide gel electrophoresis (PAGE) on both sets of samples. Without laser excitation, only G21 exhibited a smear and multiple bands (Figure 2J). This might be due to the inherent ability of G21 oligonucleotides to form G-quadruplex multimers 30. After laser excitation, all oligonucleotides displayed a clear smear phenomenon and multiple bands of different molecular weights on the PAGE. These results indicate that tightly focused laser excitation may enhance cross-linking among aggregated ssDNA, thereby increasing the quantum yield of excimer fluorescence.

Our experiments with synthetic oligonucleotides revealed that adenine-rich sequences are particularly potent generators of this red autofluorescence. This observation strongly implicates specific regions of the mitochondrial genome as the primary source of the signal. The mitochondrial D-loop, a major non-coding region, is notably rich in adenine. To confirm this link, we synthesized oligonucleotides from this region and confirmed their ability to produce a strong ~600 nm emission upon aggregation (Figure S2E-F, Table S4). Therefore, we propose that the D-loop region of mtDNA is a key contributor to the observed red autofluorescence, as its degradation releases adenine-rich fragments that form highly fluorescent excimer stacks in the acidic lysosomal environment.

### mtDNA enters the lysosome through the ESCRT-III-mediated microautophagy pathway

To investigate the mechanism by which mtDNA is translocated to lysosomes, we initially considered mitophagy as the potential route. We induced mitophagy in L929 cells using carbonyl cyanide m-chlorophenyl hydrazone (CCCP), a protonophore uncoupler which inhibits mitochondrial oxidative phosphorylation (Figure S4B). Following mitophagy induction, a significant increase in Ag-mtDNA fluorescence was detected (Figure 3A-B). Given the important impact of autophagy in cellular metabolism and the crucial role of the ATG7 gene in the autophagy process, we used an ATG7 knockout MEF cell line to abolish the autophagy pathway 31, and then induced apoptosis with the DNA alkylating agent cisplatin (Figure S4C). The results showed that the intensity of cellular red autofluorescence still increased, suggesting that the generation of red fluorescence does not solely depend on classical macroautophagy (Figure 3C-D).

As the cyclic GMP-AMP synthase (cGAS) can detect misplaced dsDNA 32 and initiate the cGAS-STING (Stimulator of Interferon Genes) innate inflammatory responses 9. The cGAS proteins could serve as effective scavengers of cytosolic DNA through affinity binding 33,34. Following cGAS-STING signaling, cGAS proteins are ubiquitinated and degraded through Endosomal Sorting Complex Required for Transport (ESCRT)-dependent microautophagy 35. Therefore, we hypothesize that microautophagy could be an alternative route for Ag-mtDNA formation. Given that Vacuolar Protein Sorting 4 (VPS4) is a key gene in the ESCRT-III-mediated endosomal microautophagy (eMI) pathway 36, we targeted VPS4 to disrupt this pathway. MDA-MB-231 cells were transfected with a plasmid expressing a VPS4 mutant to induce a dominant negative (DN) effect 37, and then cell apoptosis was induced with cisplatin (Figure S4D). The transfection reagent Lipo3000 was used as the control group to exclude the potential autofluorescence and cytotoxic effects of the reagent itself. After inhibiting cellular microautophagy with the dominant negative VPS4 mutant construct, the two-photon red autofluorescence intensity was significantly decreased (Figure 3E-F), even in cells treated with cisplatin.

It has been reported that the ESCRT-III-mediated endosomal microautophagy (eMI) pathway is coupled with the Misfolded-Protein-Associated Protein Secretion (MAPS) pathway to maintain lysosomal homeostasis, regulated by a molecular chaperone Cysteine String Protein α (CSPα, also known as DNAJC5) 37. Inhibition of the MAPS pathway leads to the accumulation of misfolded proteins, which subsequently activates the eMI pathway to compensate by promoting protein degradation. Under these conditions, Ag-mtDNA fluorescence is likely to increase. Given that Solute Carrier Family 3 Member 2 (SLC3A2, also known as CD98hc), a CSPα interactor, is essential for the MAPS pathway but not for microautophagy, it serves as a target to disrupt the MAPS pathway. To this end, we designed an shRNA targeting SLC3A2 and established an SLC3A2 knockdown cell line in MDA-MB-231 cells using a lentiviral vector (Figure S5H). Two-photon fluorescence images showed that red fluorescence intensity in SLC3A2 knockdown cells was significantly higher than that in the wild type counterparts, and further increased after cisplatin induction (Figure 3G-H, Figure S4E). This further confirmed that the formation of Ag-mtDNA fluorescence predominantly depends on the ESCRT-III-mediated eMI pathway.

### Ag-mtDNA autofluorescence as a novel reporter of programmed cell death and cellular senescence

We further investigated the characteristics of red autofluorescence in ferroptosis, necroptosis, pyroptosis (Figure S5A) as well as cellular senescence. Expression levels of the corresponding biomarkers were confirmed by RT-qPCR (Figure S5B-G) and Senescence Cell Detection Kit (Figure S5I). The lysosomes still retained the necessary acidic conditions for the denaturation of fragmented mtDNA (Figure S6A-B). Through LysoTracker Green staining and fluorescence colocalization analysis, we found that under ferroptosis, necroptosis, and pyroptosis, the two-photon red autofluorescence also colocalized with lysosomes (Figure 4A and Table 1) and the corresponding intensities were all significantly increased (Figure 4B and D-H). However, in necroptotic cells, the degree of lysosomal colocalization was reduced (Figure 4A and Table 1). Furthermore, when necroptosis was inhibited using Necrostatin-1 (Nec-1), the red fluorescence intensity was markedly decreased (Figure 4B and F). Regarding the translocation mechanisms, we also examined the expression levels of the mitophagy marker proteins Pink1 and Parkin. The results indicated that Pink1/Parkin-dependent mitophagy was barely involved in these types of PCD (Figure S6C-E), suggesting that mitochondria-released mtDNA was predominantly scavenged by the eMI pathway or the remaining Pink1/Parkin-independent mitophagy pathway like Bcl-2/E1B-19 kDa Interacting Protein 3 (BNIP3) and FUN14 Domain Containing 1 (FUNDC1) 38,39. These findings suggest that lysosomal Ag-mtDNA autofluorescence is a novel pan marker of most cell death modalities, hence emerging as a molecular imaging contrast tool to visualize the dynamics of cell death.

Similar to cell death, mitochondrial outer membrane permeabilization (MOMP) in cell senescence also releases mtDNA 10 that drives aging-related inflammation 9. We therefore suspected that the aging cell senescence process might also accumulate Ag-mtDNA in lysosomes and produce autofluorescence hallmarks. We therefore induced cellular senescence using X-ray irradiation; expectedly, the red autofluorescence intensity in senescent cells was significantly increased (Figure 4C and I) and the spectrum was consistent with those observed upon cell death induction (Figure 4J).

Aside from cell death detection, differentiating PCD types will be another critical issue in the studies of cell death dynamics. Here we explored the differential features of cells undergoing ferroptosis, necroptosis, and pyroptosis using fluorescence lifetimes and imaging morphologies. Fluorescence lifetime imaging microscopy (FLIM) revealed that the fluorescence lifetime τ1 in apoptosis, pyroptotic and necroptotic cells was significantly increased to 400-800 ps, when compared to 100-400 ps in the control counterpart. Interestingly, red fluorescence lifetimes in ferroptotic cells showed no significant change (Figure 4K-L). This reduction of fluorescence lifetime in the course of ferrotposis (Figure S7A-B) could be due to the iron ion induced hydroxyl radicals and lipid peroxidation in lysosomes 40,41, which warrants further investigation. These findings demonstrate that the intracellular morphology (Figure 4A and Figure S6F) and the fluorescence lifetime of Ag-mtDNA can readily serve as novel indicators to distinguish between distinct types of cell death.

### Autofluorescence of Ag-mtDNA can *in vivo* report aging in *C. elegans*

Mitochondrial dysfunction and cellular senescence are major hallmarks of aging that are common across organisms 42,43. Based on our finding that cell senescence produces the Ag-mtDNA autofluorescence, we wondered whether it can quantitatively reflect the aging conditions in multicellular organisms. Three strains of *C. elegans* have been used for the investigation, which are either promoting aging (*daf-16(mu86) I* mutants) 24, delaying aging (*daf-2(e1370) III* mutants) 23 and wild type (N2). The *daf-16* loss-of-function mutation results in the downregulation of key longevity-related pathways, including those involved in stress resistance, metabolism, and detoxification. This mutation significantly shortens the median lifespan of wild-type *C. elegans* and has long been recognized as a well-established model for accelerated aging. Conversely, *daf-16* is negatively regulated by the *daf-2* pathway. A mutation in *daf-2* leads to the upregulation of longevity-promoting genes which can approximately double the median lifespan.

The two-photon red autofluorescence was significantly enhanced in *daf-16(mu86) I* mutants, while it was very weak in *daf-2* mutants (Figure 5A-C and Figure S8A, C and E). In the wild type *C. elegans* (N2 group), the Ag-mtDNA autofluorescence of adult worms was significantly brighter in comparison with larval ones (Figure 5A). The FLIM results showed that the fluorescence lifetime τ1 in *daf-16(mu86) I* mutants increased to a range of 400 to 800 ps, while those in *daf-2(e1370) III* mutants and wild-type (N2) were comparatively short at 100 to 400 ps (Figure 5D-E and Figure S8B, D, F, G and H). Interestingly, a spatial heterogeneity was observed in the *daf-16* mutant, where the longer lifetime signal (blue) was predominantly localized to internal tissues, such as the intestine, while the shorter lifetime signal (more yellow) was apparent in peripheral tissues. This spatial pattern aligns with previous reports that the *daf-16/FOXO* transcription factor's role in lifespan regulation is most critical in the intestine, where its loss of function leads to the most severe aging-related cellular stress 44. Our findings indicate that increasing autofluorescence intensity and prolonged lifetime in Ag-mtDNA autofluorescence highly correlate with the aging status of *C. elegans*. Thus, the Ag-mtDNA autofluorescence reporter system provides a non-invasive measure of senescence *in vivo* and can offer valuable insights into the aging process.

### Autofluorescence of aggregated DNA can be used to detect sepsis in mice and human

The presence of circulating cell-free DNA and mitochondrial content in blood acts as a damage-associated molecular pattern (DAMP), which can trigger inflammatory responses and contribute to the progression of sepsis 45. Elevated levels of circulating cell-free DNA have been associated with the severity of sepsis and can serve as a biomarker for its diagnosis 46. Our aggregated DNA red autofluorescence is likely to be a hallmark marker for real-time monitoring and detection of sepsis. In the classical cecal ligation and puncture (CLP) model of sepsis in mice 47, at 8 h post induction, we found no significant tissue injury and damage in different organs. Bacterial load, lipopolysaccharide (LPS), and procalcitonin (PCT) levels were not significantly increased at this stage (Figure S9). After 12 h, the level of PCT showed a significant rise. At 24 h post induction, we could observe obvious organ damage, increase in plasma LPS level, and bacterial load. Since PCT is a U.S. Food and drug administration (FDA)-approved early sepsis indicator 48, we could define 8-12 h post CLP induction as the early pathology stage of sepsis before multiple organ damage occurs. In the sham control group, there were baseline intensities of aggregated DNA fluorescence emitting at a characteristic 600 nm peak (black curve in Figure 5F). We then measured the two-photon emission spectra of mouse serum at the 8^th^, 12^th^, 16^th^, and 24^th^ hour post CLP induction. The red autofluorescence in the serum of septic mice surged at the 8 h post CLP induction, which is earlier than PCT's rise. The aggregated DNA fluorescence reached a maximum at 16 h post induction (Figure 5F-G).

In a human pilot study, we compared plasma red autofluorescence intensities between sepsis patients (n = 14) and healthy controls (n = 22). The sepsis group included patients with suspected infections who met the diagnostic criteria for sepsis, defined by a Sequential Organ Failure Assessment (SOFA) score of ≥ 2 (Table S5), as verified by a physician in accordance with the Sepsis-3 definition 25. Consistent with findings from animal studies, the plasma exhibited characteristic two-photon fluorescence of aggregated DNA, peaking at 600 nm (Figure 5H). Fluorescence intensities at this wavelength were significantly higher in sepsis patients compared to healthy controls (Figure 5I), plausibly originating from degraded nucleic acid fragments released during extensive cell death caused by cytokine storms 49,50 and organ injury. This prominent feature in human clinical studies supports the potential use of aggregated DNA fluorescence as diagnostic marker for managing sepsis patients.

## Discussion and Conclusion

In this study, we demonstrate that the intriguing intracellular red autofluorescence (λ_e_~600 nm) predominantly emanates from mtDNA located in lysosomes. Our findings reveal that this autofluorescence is closely linked to the ESCRT-III-mediated microautophagy pathway, by which mtDNA enters lysosomes. Inside the acidic lysosomal lumen, ds-mtDNA undergoes denaturation hence becoming ssDNA, which exhibits significant autofluorescence under single-photon (λ_ex_ = 561 nm) or two-photon excitation (λ_ex_ = 1060 nm). Our experiments demonstrate that the red autofluorescence is highly specific to mtDNA and is primarily located within lysosomes during apoptosis, aging, and various forms of cell death, including ferroptosis, necroptosis, and pyroptosis. The colocalization of this autofluorescence with bona fide viable fluorescent lysosomal probes confirms that mtDNA plays a crucial role in generating this unique autofluorescence signal, highlighting a previously underappreciated association between mitochondrial dynamics, lysosomal function, and cellular autofluorescence.

A critical consideration for any endogenous fluorescence-based method is the potential for spectral overlap with other native cellular fluorophores. We therefore performed a rigorous assessment to ensure the specificity of the aggregated DNA signal. The most abundant sources of cellular autofluorescence, NAD(P)H and flavins, are not efficiently excited by our chosen wavelengths. NAD(P)H exhibits optimal excitation in the ultraviolet range (~350 nm) with blue emission, while flavins are best excited around 450 nm, emitting in the green-yellow spectrum 51,52. Our use of 561 nm single-photon or ≥1060 nm two-photon excitation effectively minimizes their contribution and isolates the red-shifted signal.

To further assess the specificity within the red emission window, we performed two-photon excitation-emission spectroscopy, systematically tuning the excitation wavelength from 920 nm to 1120 nm. We observed that for excitation wavelengths of 1060 nm and longer, the emission spectrum remained consistently centered at ~600 nm, which is characteristic of aggregated DNA (Figure S2G). This spectral stability strongly suggests that contributions from other known red autofluorophores, such as porphyrins (which typically have a primary emission peak near 630 nm) and bilirubin aggregates (peaking above 650 nm), are negligible under these specific excitation conditions 53,54. Taken together, these spectral analyses provide compelling evidence that aggregated DNA is the dominant molecular source of the intracellular red autofluorescence observed in our study, establishing it as a highly specific biomarker for cell death and senescence.

Further analysis using high-throughput sequencing of lysosomal DNA confirmed that the majority of the fluorescence-emitting DNA within lysosomes stemmed from mtDNA rather than from nuclear DNA. This supports the hypothesis that the lysosomal degradation of mtDNA is a critical event for the generation of this red autofluorescence. We further explored the molecular mechanism underlying this autofluorescence, hypothesizing that it might arise from DNA excimer formation, particularly due to the unique structural properties of DNA under the acidic conditions present in lysosomes. Our experimental evidence suggests that ssDNA, particularly adenine-rich oligonucleotide sequences, most significantly contribute to the observed autofluorescence. This is consistent with the fluorescence emission spectra and fluorescence lifetime of various DNA oligonucleotides, which show the most intense autofluorescence at ~ 600 nm when excited by 1060 nm using a two-photon microscope. It is important to note that this autofluorescence also appears in unprogrammed cell death. Our previous work demonstrated that primary necrosis, induced by H₂O₂, generates a similar ~600 nm emission peak 16. However, necrosis is clearly distinguishable by two key features: a homogeneous intracellular distribution of the signal due to lysosomal rupture, and a significantly decreased fluorescence lifetime. This autofluorescence was established not only in cell death *in vitro* but also in aging models *in vivo*. In an aging *C. elegans* model, adult worms exhibited significantly enhanced red autofluorescence compared to larvae. An enhanced aging *C. elegans* strain exhibited a much higher red autofluorescence than wild type worms. These results suggest that this autofluorescence could serve as a *bona fide* biomarker of aging.

Similarly, in a CLP sepsis mouse model and a human pilot study, the sera of septic mice (with high PCT levels) and sepsis patients (SOFA ≥ 2) displayed a significantly enhanced autofluorescence at 600 nm. As previously shown, the aggregated DNA likely stems from degraded extrachromosomal circular DNA elements (eccDNAs) released by apoptotic cells 49 or from neutrophil extracellular traps (NETs) formed during neutrophil NETosis 50. These aggregates are potent inducers of cytokine storms 49 and disseminated intravascular coagulation (DIC) 50,55, contributing to sepsis pathology. The increase in aggregated DNA fluorescence could serve as a biomarker to guide anti-cytokine storm or anti-DIC therapies in sepsis management. The origin of this circulating aggregated DNA in the bloodstream is likely multifaceted. Beyond passive release from necrotic cells, these nucleic acid fragments are often actively packaged and transported within extracellular vesicles (EVs). These EVs act as key carriers of damage-associated molecular patterns (DAMPs), facilitating intercellular communication and propagating systemic inflammation in sepsis. Intriguingly, the field of nanomedicine is now harnessing these same biological nanoparticles, engineering them to create a new class of targeted nanomedicines for therapeutic delivery 56. This dual nature of EVs—as both mediators of pathology and potential therapeutic vehicles—suggests a promising future direction: analyzing the Ag-DNA fluorescence specifically within the EV fraction isolated from plasma could offer a more sensitive and mechanistically refined biomarker for tracking sepsis progression and therapeutic response.

In our previous studies 16,17, we also observed that this characteristic red autofluorescence could serve as an endogenous reporter for 3D pharmacodynamic monitoring in vibratome-sliced animal and patient-derived tumor organoids under fresh, unfixed conditions. Compared to conventional methods, this intrinsic fluorescence enables real-time assessment of drug responses in three dimensions, eliminating the need for fixation and exogenous dye staining. The ability of this unique autofluorescence to reliably report on the pathophysiological state of cells and whole organisms could pave the way towards non-invasive imaging and diagnostic techniques. Beyond cancer and sepsis, this biomarker holds promise for monitoring inflammatory autoimmune diseases such as Rheumatoid Arthritis, where cell death in synovial joints drives pathology, or Multiple Sclerosis, where neuronal cell death in the central nervous system is a key hallmark of disease progression. Future studies should focus on further elucidating the molecular mechanisms underlying this autofluorescence and realizing its full potential in these diverse biomedical applications. These findings highlight the potential of aggregated DNA fluorescence as a valuable theranostic tool for various pathophysiological states.

While the field of advanced biomedical imaging is rapidly advancing through the design of sophisticated synthetic agents, such as biodegradable bismuth-based nanotheranostics for multimodal CT/MR imaging and photothermal therapy 57, our discovery provides a powerful and complementary paradigm. By harnessing an entirely endogenous, label-free biomarker, our approach circumvents the inherent challenges associated with the synthesis, systemic delivery, biocompatibility, and potential long-term toxicity of exogenous probes. The ability to directly visualize a fundamental biological process like cell death using the cell's own components offers an unparalleled and unperturbed window into native pathophysiology. This positions Ag-mtDNA fluorescence not merely as a diagnostic tool, but as a foundational method for real-time, personalized monitoring of disease dynamics and therapeutic efficacy, paving the way for truly non-invasive biological insights.

In conclusion, our current study identified a novel autofluorescence property of mtDNA within lysosomes that could be readily harnessed as a biomarker for various forms of cell death, cellular aging, and pathophysiological systemic conditions such as sepsis. The ability of this unique autofluorescence to reliably report on the pathophysiological state of cells and whole organisms could pave the way towards non-invasive imaging and diagnostic techniques, including aging research as well as malignant and non-malignant disease monitoring. Future studies should focus on further elucidating the molecular mechanisms underlying this autofluorescence and realizing its full potential in various biomedical applications.

## Supplementary Material

Supplementary figures and tables, methods.

## Figures and Tables

**Figure 6:**
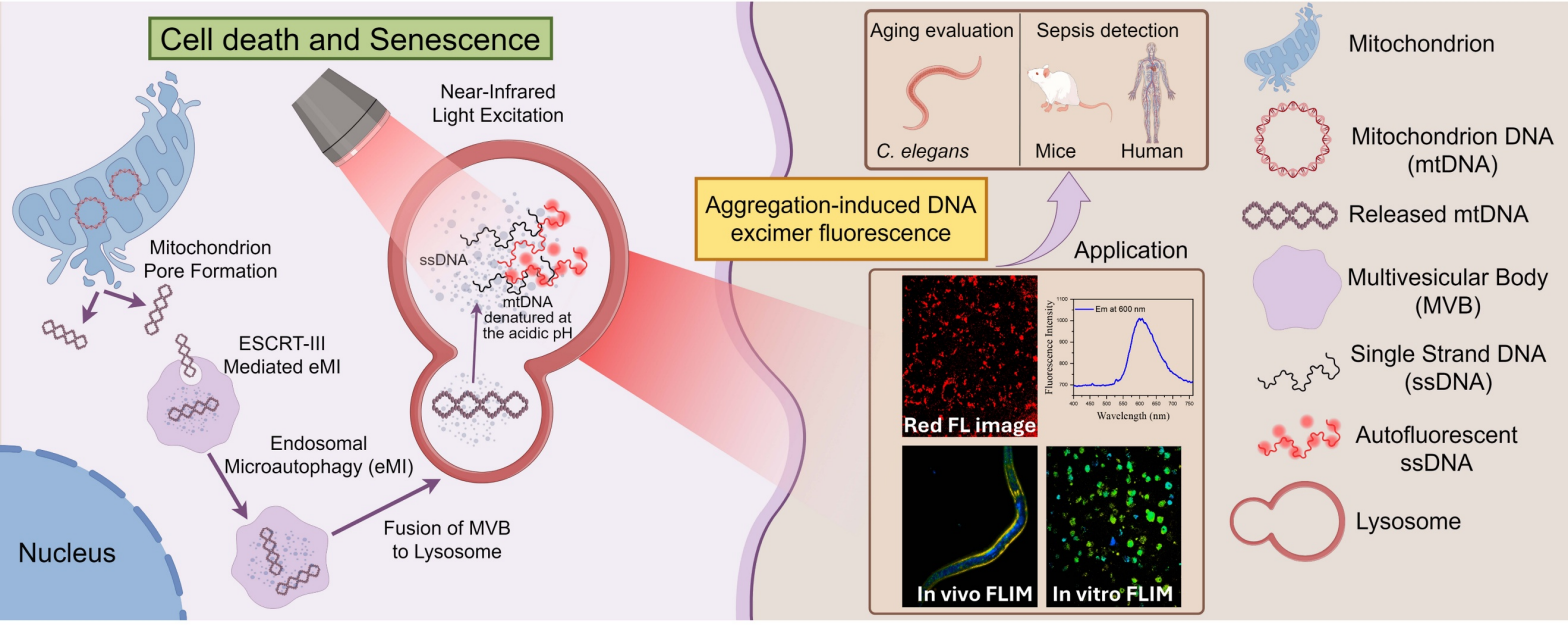
**Schematic representation of the mechanism underlying cell autofluorescence and its application.** (By Figdraw)**.** During cell death or aging, mitochondrial pores form, releasing mitochondrial DNA into the cytoplasm. The mitochondrial DNA in the cytoplasm is transported to the lysosome via ESCRT-III-mediated microautophagy. In the acidic environment of the lysosome, the mitochondrial DNA denatures and aggregates, which can emit red autofluorescence when excited by near-infrared light.

**Table 1:** Pearson and Manders coefficients obtained from colocalization analysis with ImageJ coloc2.

	Pearson's R value	Manders' tM1	Manders' tM2
Apoptosis	0.96	0.981	0.982
Ferroptosis	0.83	0.964	0.964
Pyroptosis	0.92	0.977	0.979
Necroptosis	0.62	0.828	0.663
Mitophagy	0.81	0.922	0.925

## Abbreviations

PCD: Programmed Cell Death
MOMP: Mitochondrial Outer Membrane Permeabilization
mtDNA: Mitochondrial DNA
SASP: Senescence-Associated Secretory Phenotype
Ag-mtDNA: Aggregated mitochondrial DNA
AIE: Aggregation-Induced Emission
ssDNA: Single-stranded DNA
dsDNA: Double-stranded DNA
FLIM: Fluorescence Lifetime Imaging Microscopy
CLP: Cecal Ligation and Puncture
LPS: Lipopolysaccharide
PCT: Procalcitonin
FDA: Food and Drug Administration
SOFA: Sequential Organ Failure Assessment
DAMPs: Damage-Associated Molecular Patterns
eccDNAs: Extrachromosomal circular DNA elements
NETs: Neutrophil Extracellular Traps
DIC: Disseminated Intravascular coagulation
RIPK: Receptor-Interacting Protein Kinase
cGAS: Cyclic GMP-AMP synthase
STING: Stimulator of Interferon Gene
PI: Propidium Iodide
NAD(P)H: Nicotinamide Adenine Dinucleotide (Phosphate), reduced form
SHG: Second-Harmonic Generation
ESCRT: Endosomal Sorting Complex Required for Transport
eMI: endosomal Microautophagy
TCSPC: Time-Correlated Single-Photon Counting
ATCC: American Type Culture Collection
MEF: Mouse Embryonic Fibroblast
ATG7: Autophagy Related 7
DMEM: Dulbecco's Modified Eagle Medium
FBS: Fetal Bovine Serum
TNF: Tumor Necrosis Factor
CCCP: Carbonyl cyanide m-chlorophenyl hydrazone
PMT: Photomultiplier Tube
IRF: Instrument Response Function
FSC: Forward Scatter
SSC: Side Scatter
RT-qPCR: Real-Time Quantitative Polymerase Chain Reaction
SLC3A2: Solute Carrier Family 3 Member 2
MAPS: Misfolded-protein-Associated Protein Secretion
shRNA: short hairpin RNA
CSPα: Cysteine String Protein α
BNIP3: BCL2/adenovirus E1B 19 kDa protein-interacting protein 3
FUNDC1: FUN14 Domain Containing 1
daf: abnormal DAuer Formation
CGC: Caenorhabditis Genetics Center
SPF: Specific Pathogen-Free
SEM: Standard Error of the Mean
