# Supplementary material for: Aggregation-induced emission of DNA fluorescence as a novel pan-marker of cell death, senescence and sepsis in vitro and in vivo
Source: Theranostics. 2026 Jan 1;16(2):1063–81. doi: 10.7150/thno.122009 (PMC12675146; doi:10.7150/thno.122009)
Supplement: Supplementary file 1 — Supplementary figures and tables, methods. [file thnov16p1063s1.pdf]

Supplementary Information

Aggregation-induced emission of DNA fluorescence as a novel pan-marker of cell death, senescence and sepsis *in vitro* and *in vivo*

Yiling Hu, Xiaoyan Wang, Ke Lu, Chonin Cheang, Yichen Liu, Yaru Zhu, Liming Xie, Moxin Li, Qingqing Zhou, Wenshan Yan, Ying Hou, Hongjie Zhang, Weiming Zheng, Changhuo Xu, Xiuping Chen, Han-Ming Shen, Zhen-Ju Huang, Yi-Hao Chen, Yi Zhou, Pei-Lin Cai, Hao Zhong, Lu Liu, Xi-Ping Wu, Xingxing Liu, Xiaolin Li, Ning Zeng,Yehuda G. Assaraf, and Tzu-Ming Liu

Contents

■ Supplementary Figures

Supplementary Figure 1. Imaging red autofluorescence in cells and DNA oligos.
Supplementary Figure 2. Photophysical properties of aggregated DNA fluorescence.
Supplementary Figure 3. Lysosomal DNA sequencing confirms mitochondrial DNA enrichment across various cell death and senescence models.
Supplementary Figure 4. The scatter plots of Annexin V/PI flow cytometry.
Supplementary Figure 5. Biochemical assays confirming the success induction of cell death and senescence.
Supplementary Figure 6. Lysosome's pH value, autophagy activity, and intracellular morphology of red autofluorescence in various types of cell death.
Supplementary Figure 7. Time-dependent lifetime of ferroptosis and Images of blank agarose gel and control lysosome sample.
Supplementary Figure 8. In vivo bright-field images, two-photon red autofluorescence images, and τm, τ1, τ2 lifetime imaging microscopy images of C. elegans.
Supplementary Figure 9. Blood tests in sepsis animal model.
Supplementary Figure 10. The image processing interface and steps in ImageJ software.
Supplementary Figure 11. The lifetime image processing interface of SPCImage software.
Supplementary Figure 12. Fluorescence images and corresponding standard curve of pHLys Red.
Supplementary Table 1. Fluorescence Quantum Yield of Aggregated A21 Oligonucleotide.
Supplementary Table 2. Design of DNA oligos with various sequences.
Supplementary Table 3. The longest consecutive match rate of the oligos to mtDNA.
Supplementary Table 4. D-loop sequence of human mtDNA
Supplementary Table 5. Clinical characteristics of sepsis patients recruited in this study.

■ Supplementary Methods

1. Two-Photon Fluorescence Image Analysis and Spectral Data Acquisition
2. Analysis of two-photon red autofluorescence lifetime in a cell
3. Evaluating colocalization between lysosomes and Ag-mtDNA
4. Analysis of cell death by Annexin V/PI flow cytometry

5. Determination of lysosomal pH values in cell death
6. Analysis of next generation sequencing
7. Photophysical Characterization of Aggregated DNA
8. Preparation of Oligonucleotide Solutions
9. Clinical study

Supplementary Figures

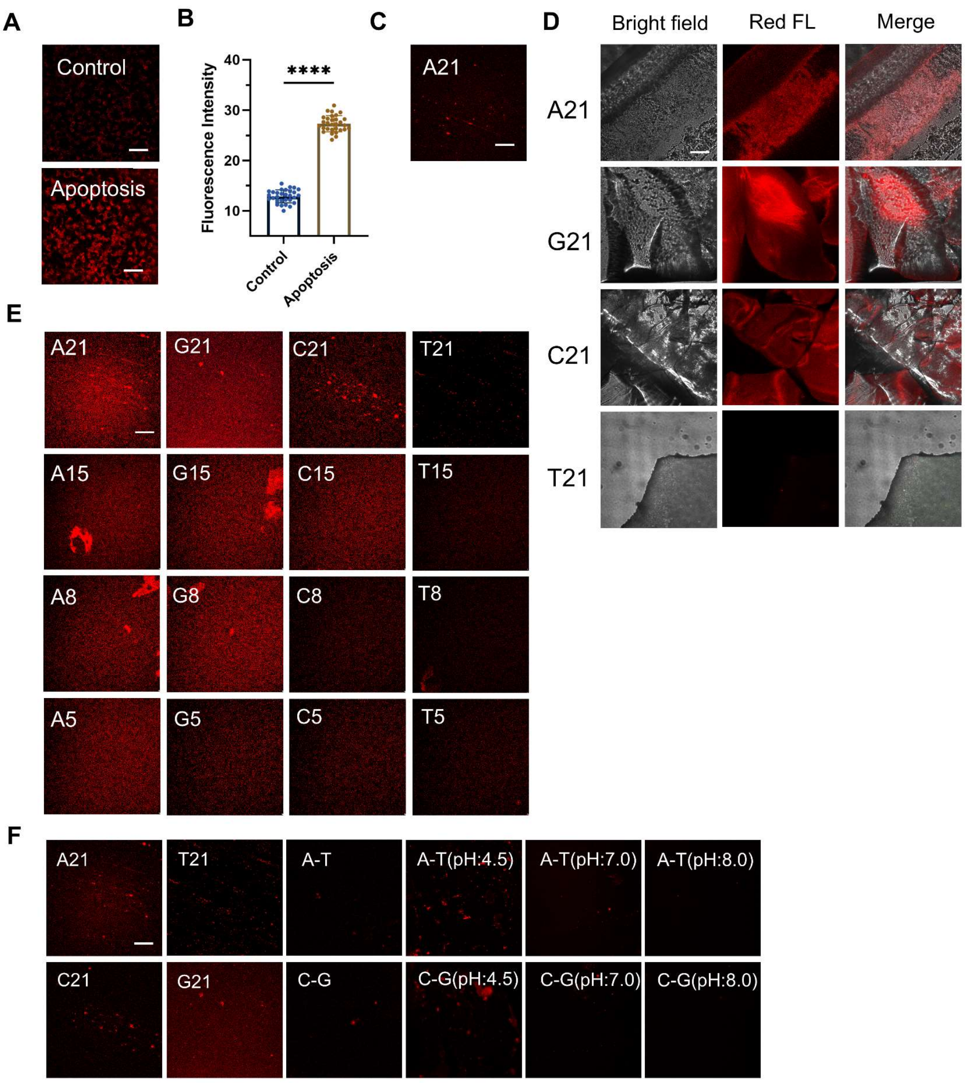

Supplementary Figure 1. Imaging red autofluorescence in cells and DNA oligonucleotides. (**A-C**) Single photon excited (λ_{ex} = 561 nm) confocal red autofluorescence images of (**A**) L929 cells and (**C**) polyA oligonucleotides (A21, 100 μM in ddH_2O). Objective NA = 1.15. (**D** and **E**) Two-photon (λ_{ex} = 1060 nm) red fluorescence images of (**D**) solid single-stranded oligos composed of 21 bases and (**E**) 100 μM single-stranded oligo DNA solution with varying base lengths. (**F**) Two-photon (λ_{ex} = 1060 nm) red fluorescence images of the 21-base oligo after annealing into double strands based on A-T and C-G base pairing, followed by denaturation into single strands using hydrochloric acid. A, T, C, and G represent adenine, thymine, cytosine, and guanine, respectively. Error bars represent the mean ± SD, ****: $p < 0.0001$, (n = 30). (All scale bars: 50 μm)

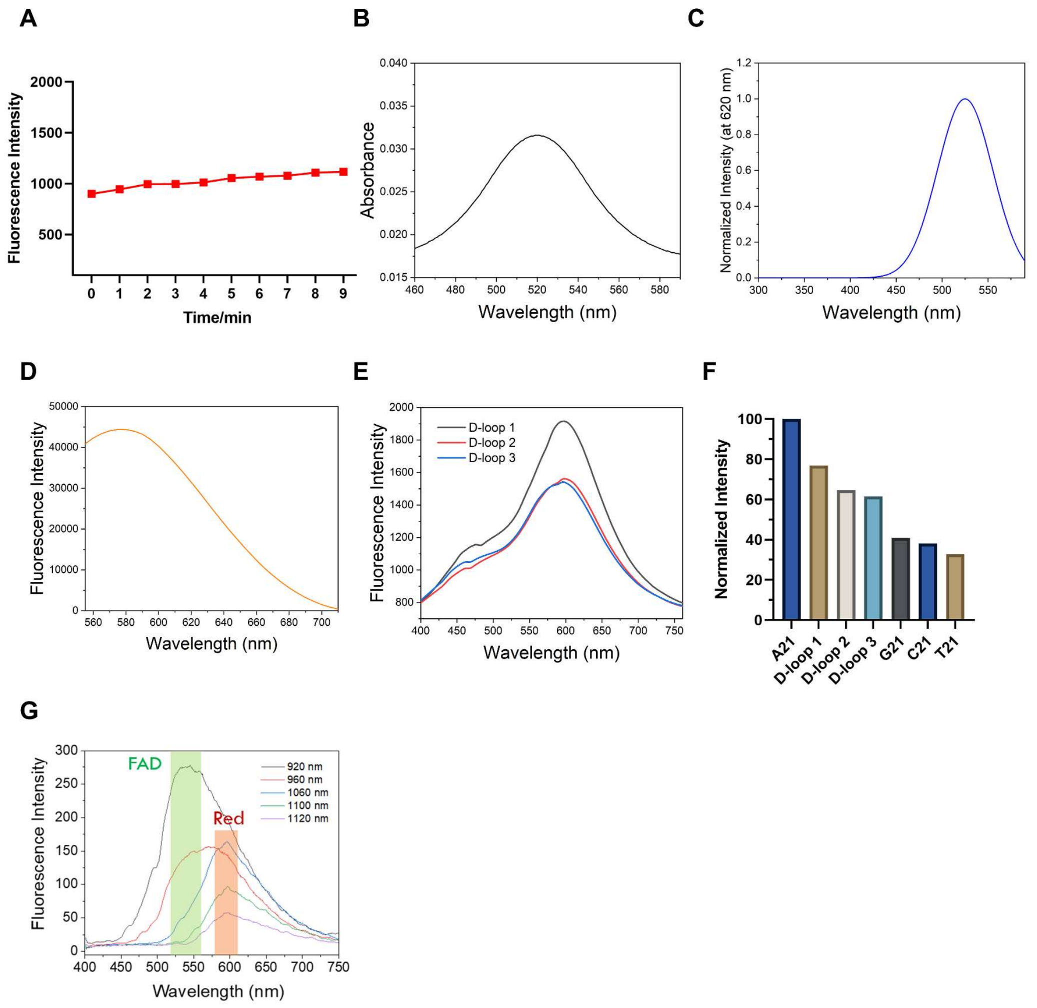

Supplementary Figure 2. Photophysical properties of aggregated DNA fluorescence. (**A**) Photostability analysis of aggregated 100 μM polyA (A21) oligonucleotides. Fluorescence intensities were recorded after continuous two-photon excitation (1060 nm) for the indicated durations (0 to 9 min). (**B**) Absorption spectrum of 1 mM polyA (A21) oligonucleotides in aqueous solution, indicating a broad absorption band in the visible range of 460-590 nm. (**C**) Fluorescence excitation spectrum of aggregated polyA (A21). The emission was monitored at 620 nm while the excitation wavelength was scanned. The spectrum reveals a broad excitation range, primarily concentrated between 450 nm and 600 nm. (**D**) Single-photon fluorescence emission spectrum of aggregated polyA (A21) upon excitation at 530 nm. The emission was collected from 555 nm to 710 nm and shows the characteristic emission peak centered at ~600 nm. (**E-F**) Two-photon emission spectrum and corresponding fluorescence intensity quantification of an aggregated oligonucleotide sequence derived from the human mitochondrial D-loop compared with A21, T21, C21 and G21, excited at 1060 nm. (**G**) Representative fluorescence emission spectra from apoptotic cells were recorded under two-photon excitation at various wavelengths, as indicated in the legend (from 920 nm to 1120 nm).

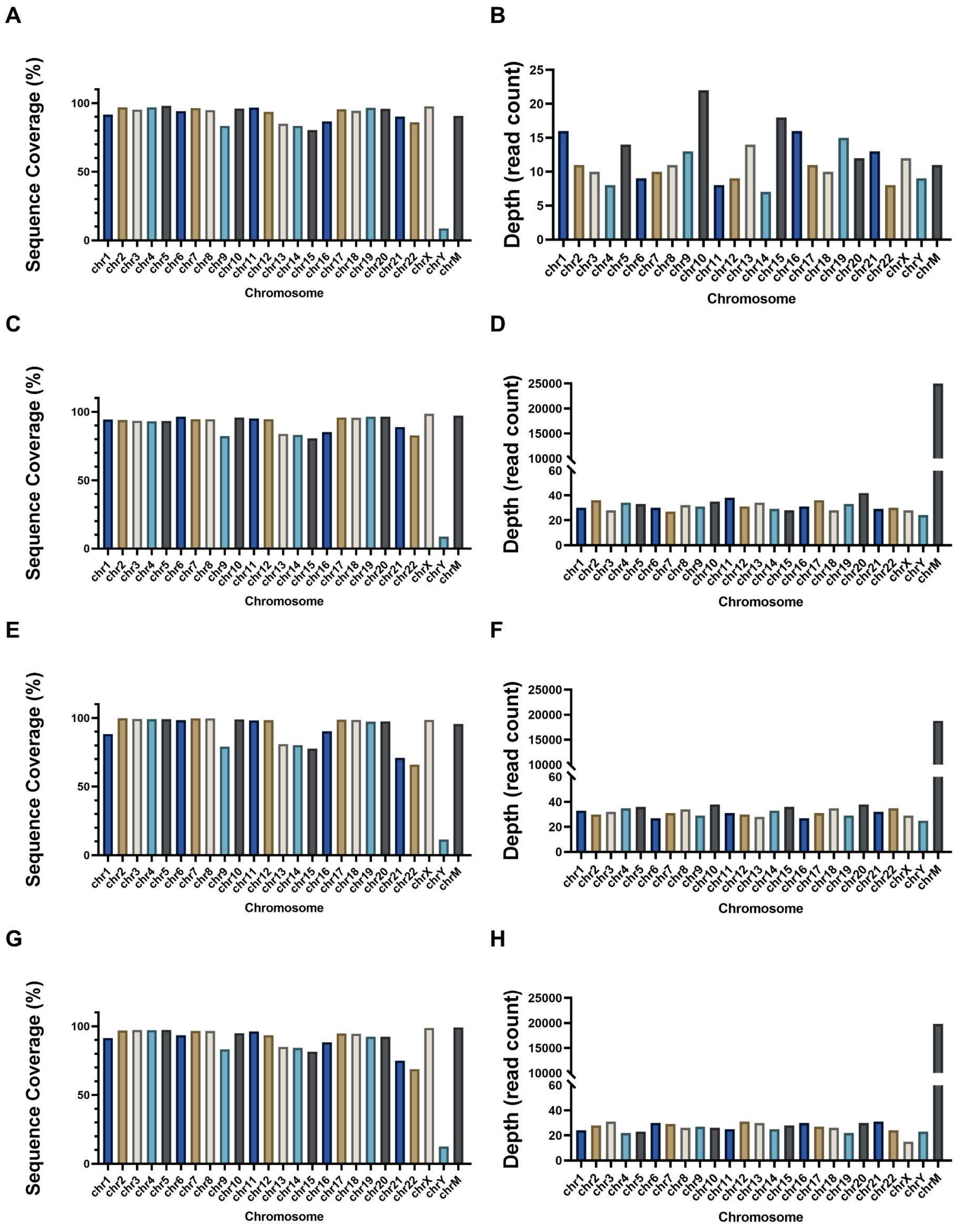
A
B
C
D
E
F
G
H
Sequence Coverage (%)
Depth (read count)
Chromosome
chr1 chr2 chr3 chr4 chr5 chr6 chr7 chr8 chr9 chr10 chr11 chr12 chr13 chr14 chr15 chr16 chr17 chr18 chr19 chr20 chr21 chr22 chrX chrY chrM
0
50
100
5
10
15
20
25
40
60
10000
15000
20000
25000

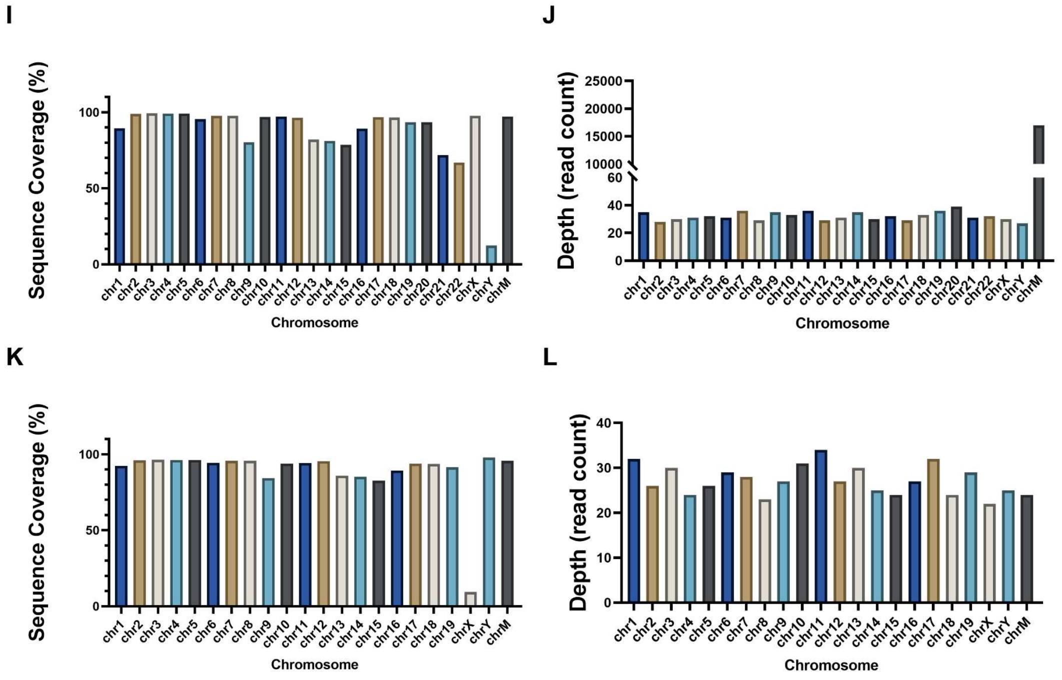

Supplementary Figure 3. Lysosomal DNA sequencing confirms mitochondrial DNA enrichment across various cell death and senescence models. High-throughput sequencing results showing (**A, C, E, G, I, K**) genome coverage and (**B, D, F, H, J, L**) sequencing depth of lysosome-aggregated DNA in (**A-J**) MDA-MB-231 cells under (**A, B**) control, (**C, D**) mitophagy, (**E, F**) necroptosis, (**G, H**) ferroptosis, (**I, J**) pyroptosis, and (**K, L**) L929 cells under senescence. The chromosomal origin of fragments was aligned with the complete cellular genome. ChrM denotes mitochondrial DNA.

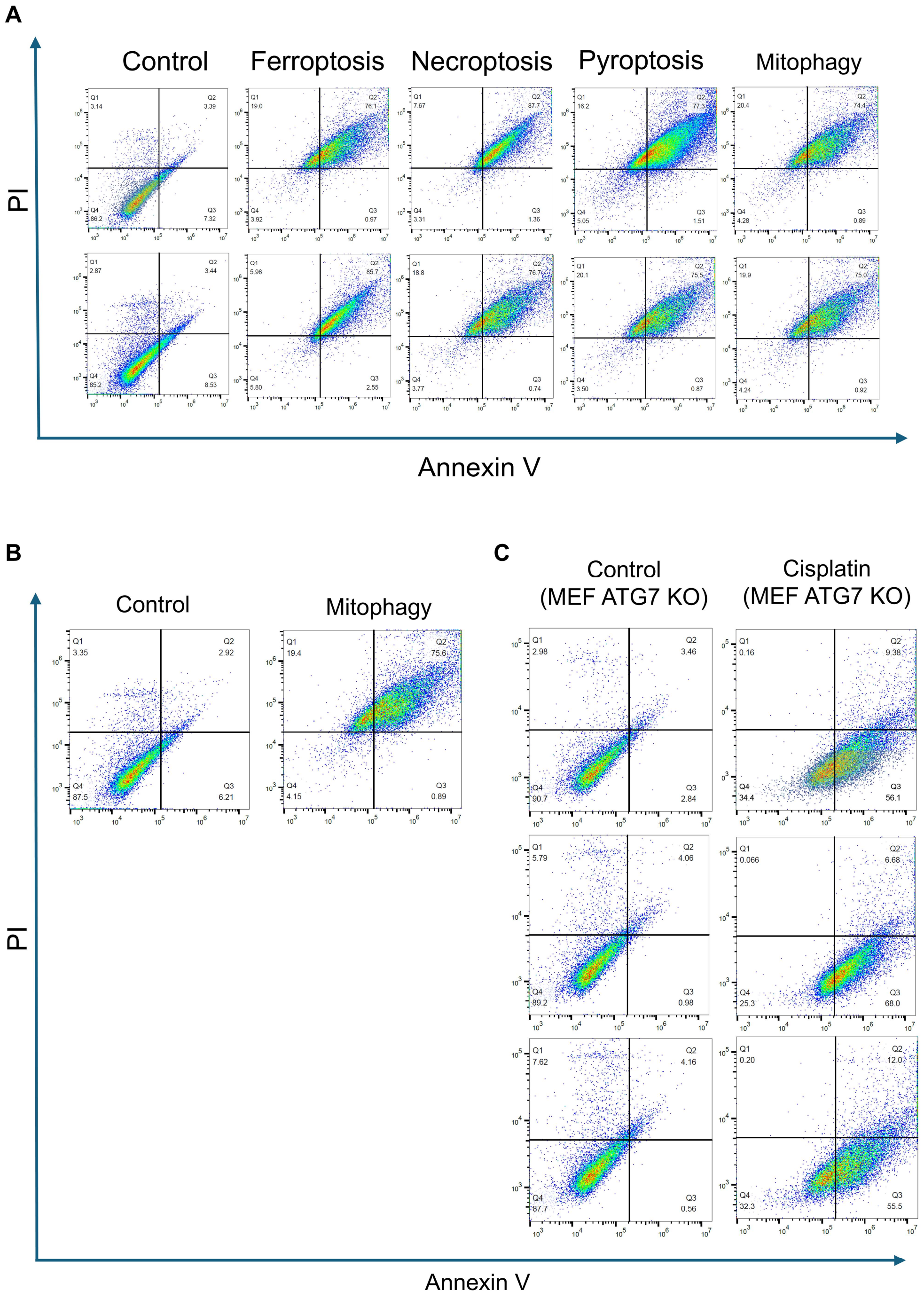
A
Control
Ferroptosis
Necroptosis
Pyroptosis
Mitophagy
PI
Annexin V
B
Control
Mitophagy
PI
C
Control
(MEF ATG7 KO)
Cisplatin
(MEF ATG7 KO)
Annexin V

D

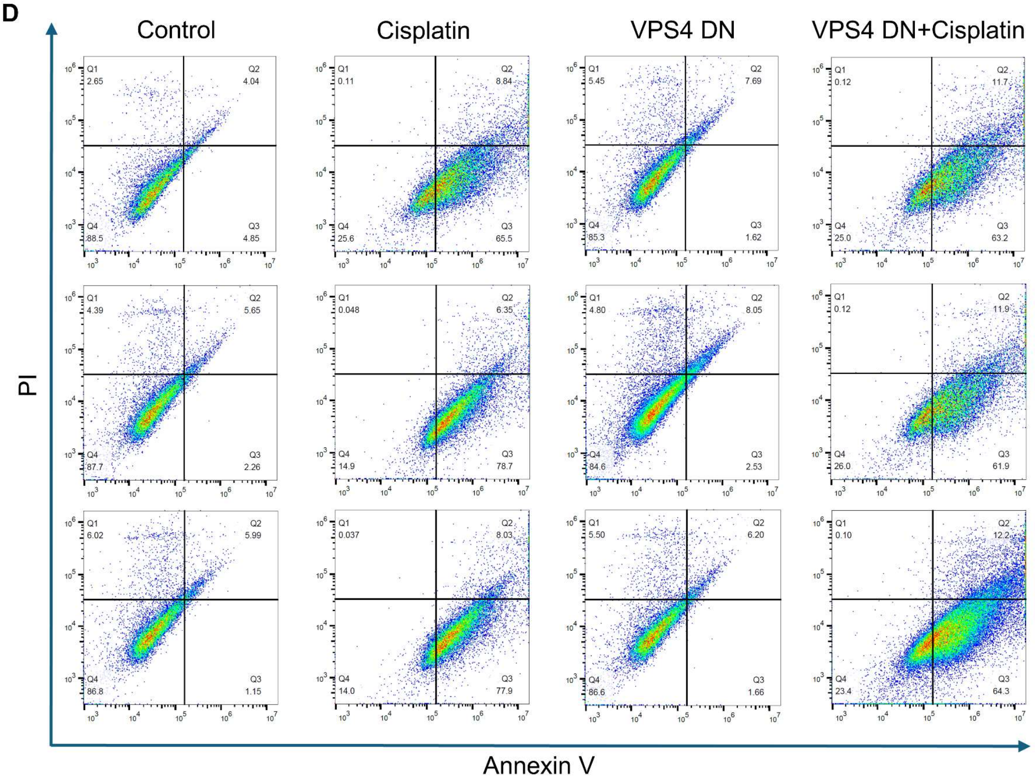

E

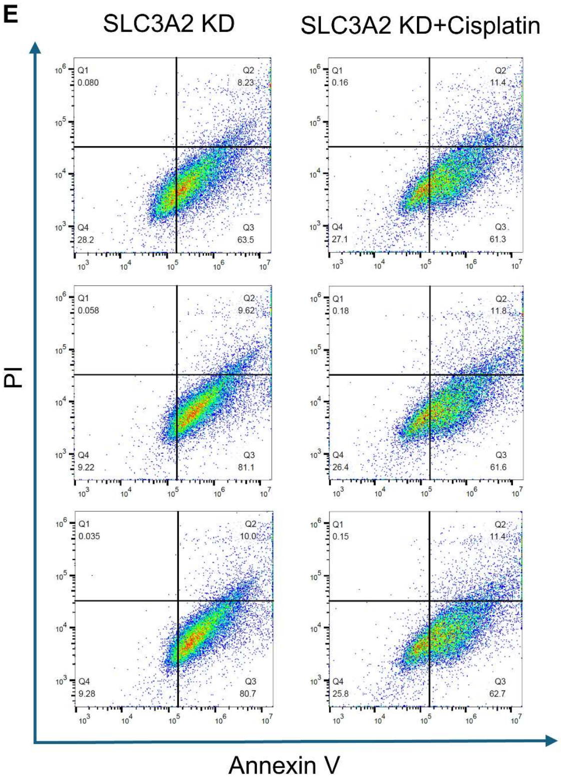

Supplementary Figure 4. The scatter plots of Annexin V/PI flow cytometry, comparing control groups with (**A**) ferroptosis, necroptosis, pyroptosis and mitophagy of L929 cells, (**B**) CCCP-induced mitophagy of L929 cells, cisplatin-induced apoptosis of (**C**) ATG7 knock out MEF cells, (**D**) VPS4 dominant negative (DN) MDA-MB-231 cells, and **(E)** SLC3A2 knock down (KD) MDA-MB-231 cells.

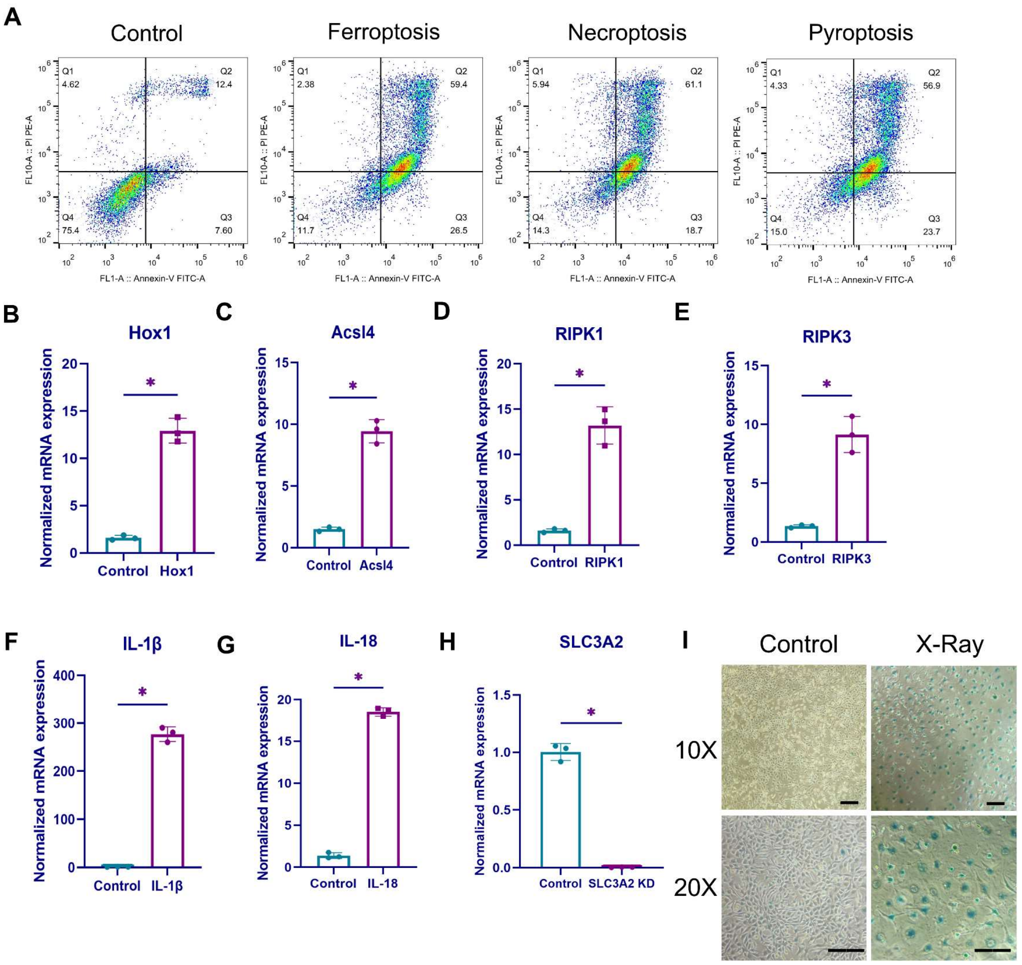

Supplementary Figure 5. Biochemical assays confirming the success induction of cell death and senescence. (**A**) Flow cytometry analysis of cell death status using annexin V/PI staining. (**B** to **G**) Real-time qPCR detection of (**B** and **C**) ferroptosis marker genes Hox1 and Acsl4 expression, (**D** and **E**) necroptosis marker genes RIPK1 and RIPK3 expression, and (**F** and **G**) pyroptosis marker genes IL-1β and IL-18 expression. (**H**) Expression of SLC3A2 in control cells and SLC3A2 knock down cells (n=3). (**I**) Images of X-ray-induced cellular senescence detected using a β-Galactosidase activity assay kit, taken under optical microscope with 10× and 20× objectives. Scale bar: 100 µm, error bars represent the mean ± SD, *: p-value < 0.05.

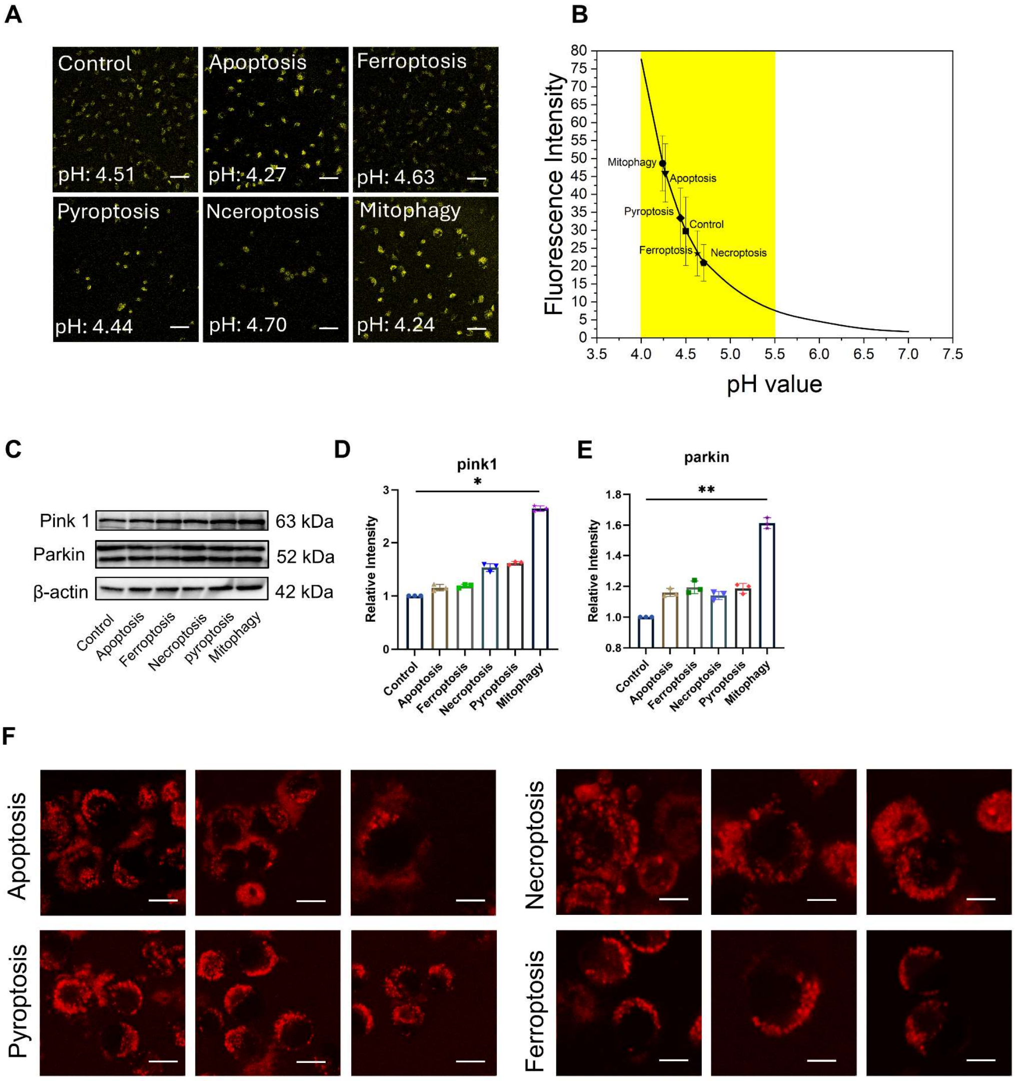

Supplementary Figure 6. Lysosome's pH value, autophagy activity, and intracellular morphology of red autofluorescence in various types of cell death. (**A** and **B**) Quantification of lysosome's pH levels in various programmed cell death types by measuring the fluorescence intensity of pHLys Red probes (λ_{ex} = 561 nm, λ_{em} = 570-620 nm) in wild type L929 cells. Using reference samples with pH values at 4.0, 4.5, 5.0, 5.5, 6.0, 6.5, and 7.0, a standard calibration curve was generated for calculating pH values from fluorescence intensity. Scale bars: 50 µm. (**C** to **E**) Expression levels of mitophagy marker proteins PINK1 and Parkin during various forms of programmed cell death. (**F**) Morphological images of two-photon (λ_{ex} = 1060 nm) red autofluorescence in cells during different types of programmed cell death. Scale bars: 10 µm, error bars represent the mean ± SD, *: *p*-value < 0.05, **: *p*-value < 0.01.

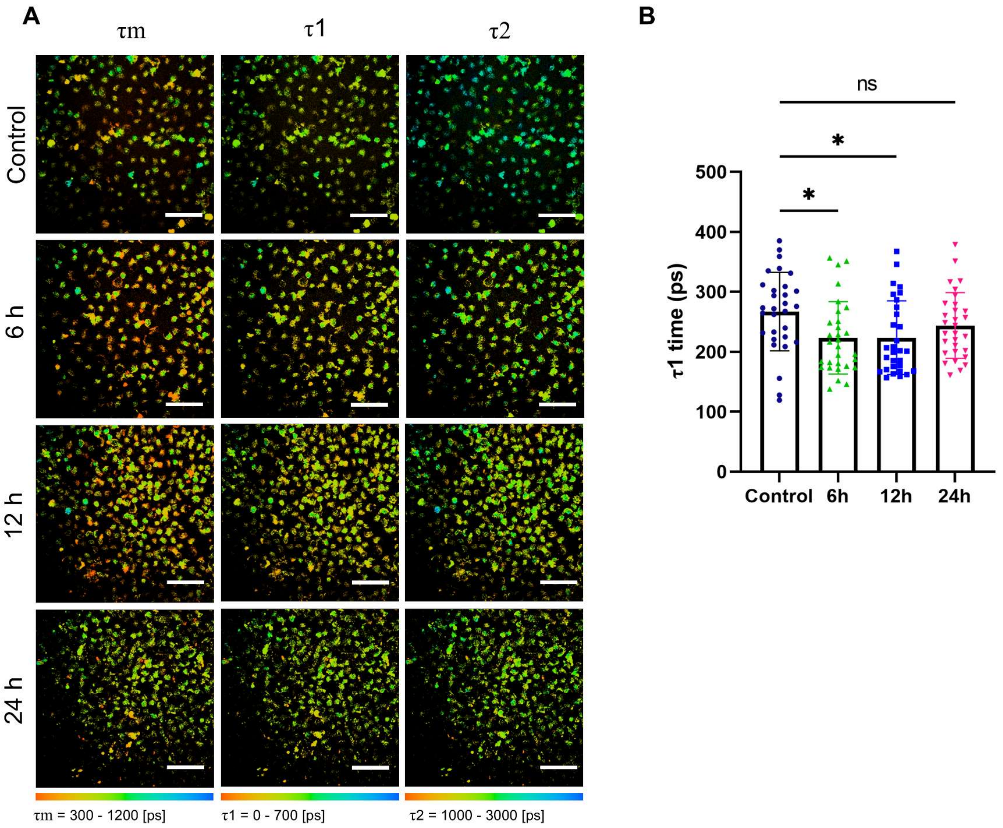

Supplementary Figure 7. Time-dependent fluorescence lifetime imaging microscopy of ferroptosis cells. (**A**) Fluorescence lifetime microscopy images and (**B**) τ1 time of L929 cells (n = 30) before (control) and after (6h, 12h, and 24h) ferroptosis induction. Scale bars: 50 µm. ns: not significant, error bars represent the mean ± SD, *: $p<0.05$.

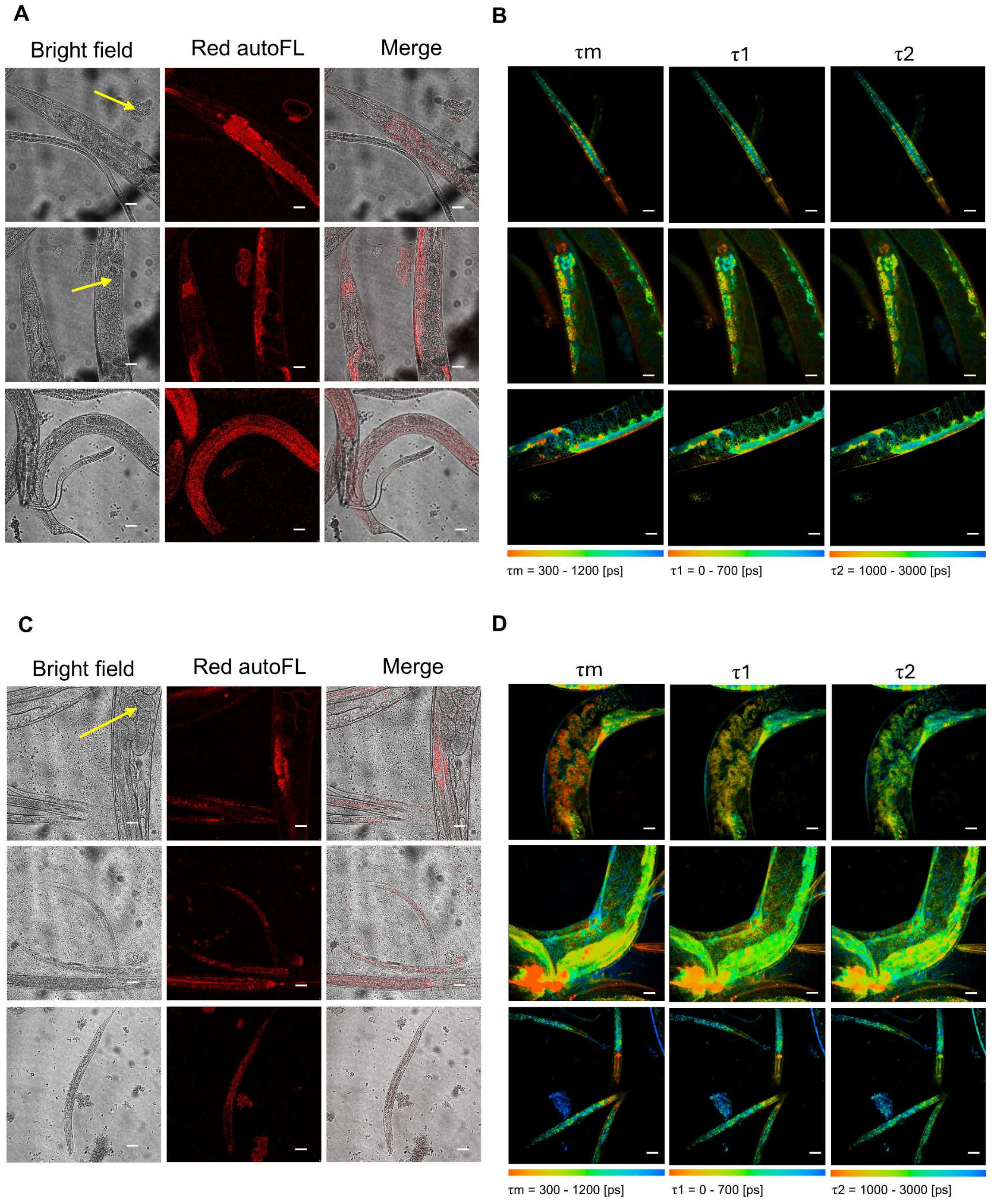
A
Bright field
Red autoFL
Merge
B
τm
τ1
τ2
τm = 300 - 1200 [ps]
τ1 = 0 - 700 [ps]
τ2 = 1000 - 3000 [ps]
C
Bright field
Red autoFL
Merge
D
τm
τ1
τ2
τm = 300 - 1200 [ps]
τ1 = 0 - 700 [ps]
τ2 = 1000 - 3000 [ps]

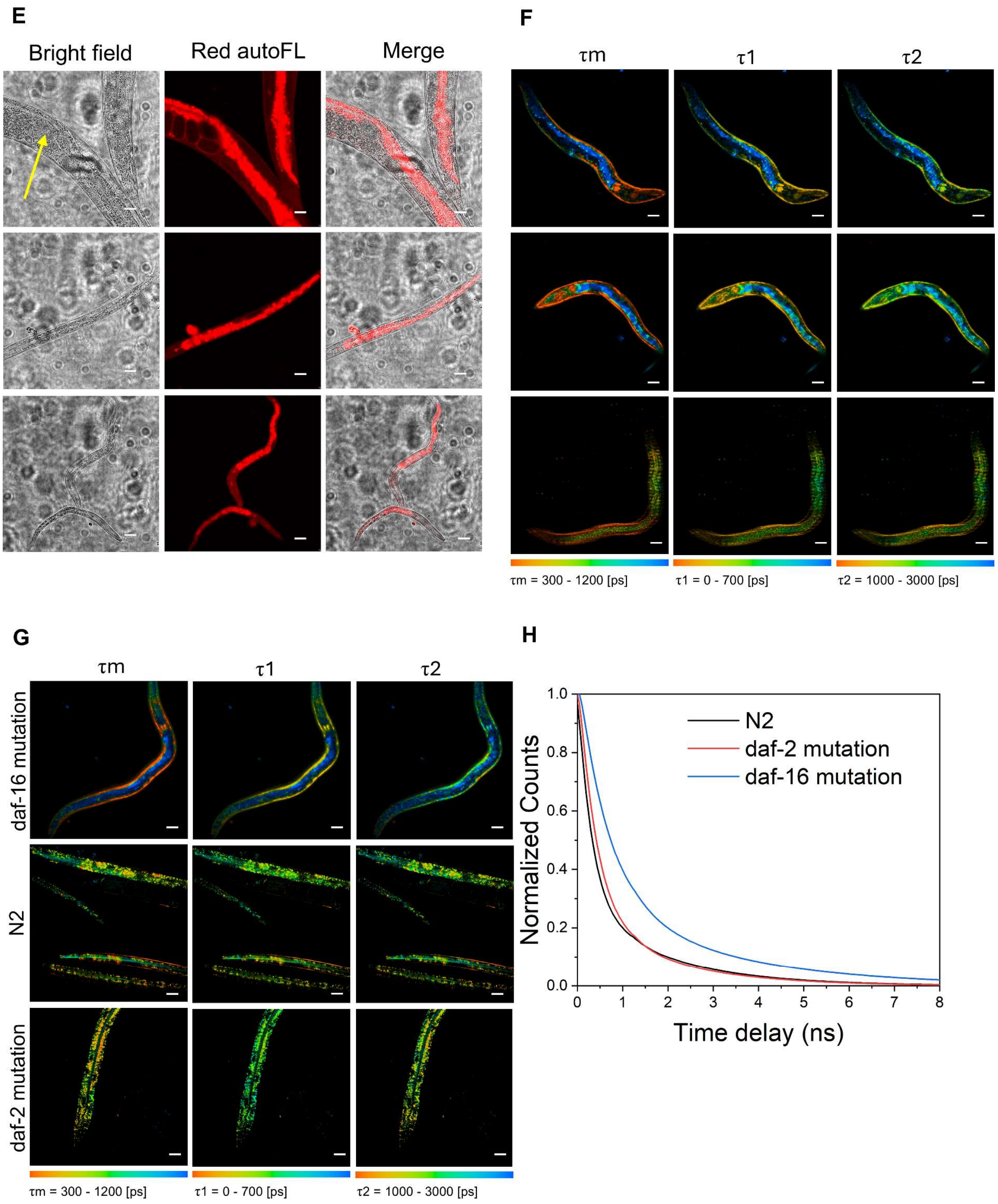

Supplementary Figure 8. *In vivo* bright-field images, two-photon red autofluorescence images, and τm, τ1, τ2 lifetime imaging microscopy of (**A** and **B**) wild-type N2, (**C** and **D**) aging-delayed *daf-2* mutation, and (**E** and **F**) aging-promoted *daf-16* mutation *C. elegans*. (**G**) fluorescence lifetime imaging microscopy and (**H**) lifetime traces of two-photon red autofluorescence in three *C. elegans* strains mentioned above. λ_{ex} = 1060 nm, Scale bars: 50 µm.

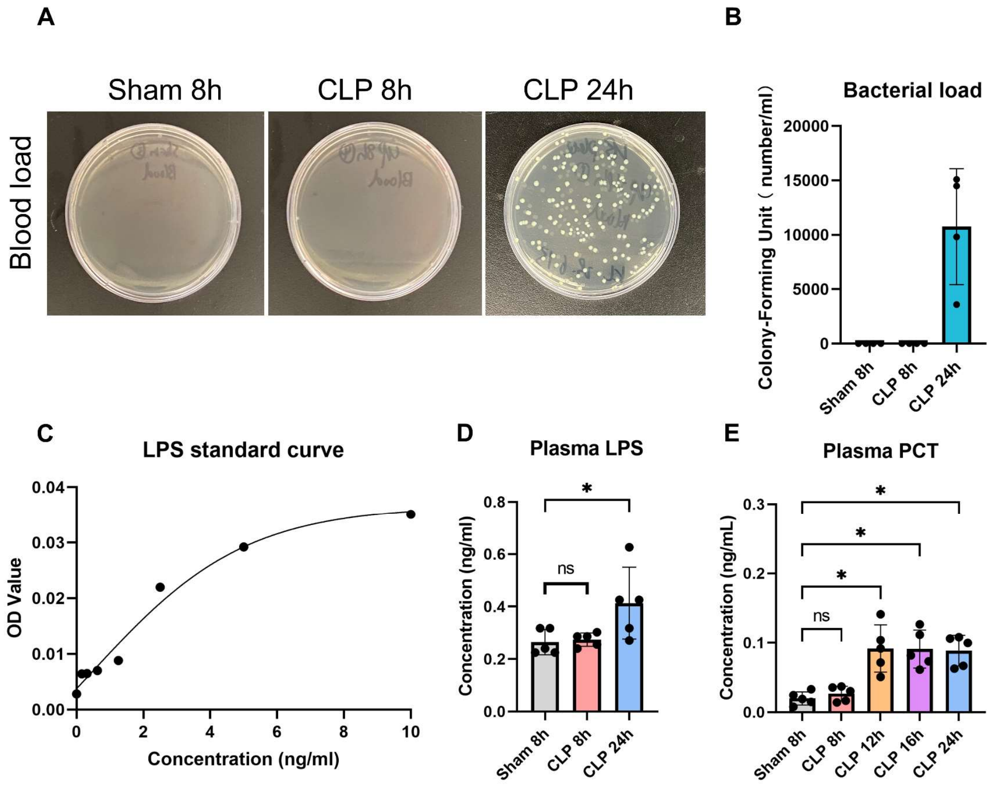

Supplementary Figure 9. Blood tests in sepsis animal model. (**A**) Blood culture images and (**B**) corresponding bacterial load (n = 4). (**C**) The calibration curve for the enzyme-linked immunosorbent assay (ELISA) of plasma PCT levels. (**D**) Plasma lipopolysaccharide (LPS) levels (n = 5), and (**E**) plasma procalcitonin (PCT) levels (n = 5) of sepsis mice measured at 8 h, 12 h, 16 h, and 24 h post induction of cecal ligation puncture (CLP). Mice in sham control group do not perform cecal ligation puncture. ns: not significant, error bars represent the mean ± SD, *: *p*-value < 0.05.

Supplementary Methods

1. Two-Photon Fluorescence Image Analysis and Spectral Data Acquisition

The imaging conditions of the DNA fluorescence images are 1060 nm excitation wavelength, 1024 × 1024 pixel number, 1.1 μs pixel dwell time, 10 mW laser power after the objective, 2 frames average for noise suppression, and 220 V bias voltage.

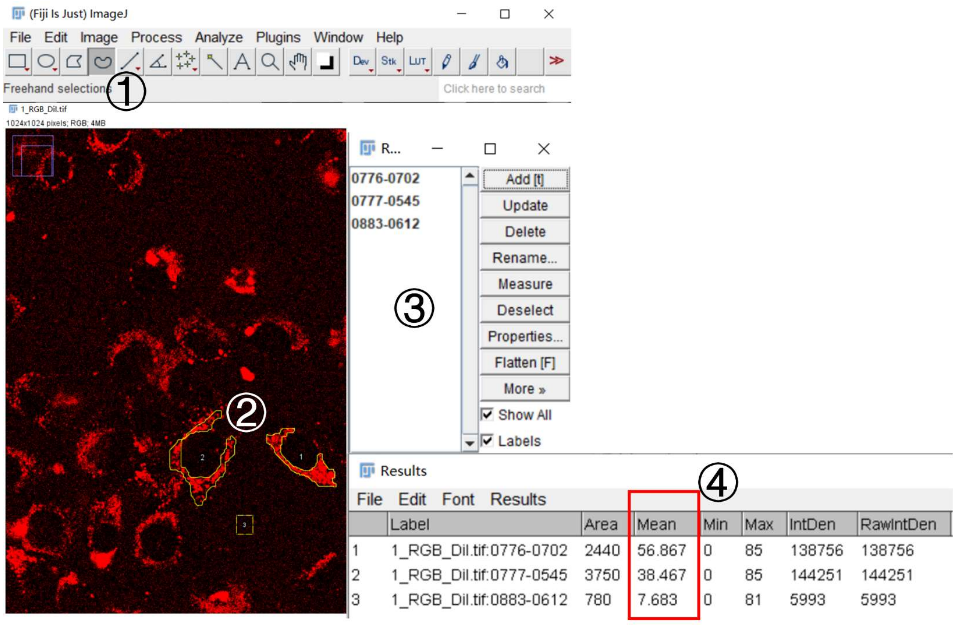

Supplementary Figure 10. The image processing interface and steps in ImageJ software.

The image processing steps:

① Import the two-photon autofluorescence image of cells into the ImageJ software and enable the freehand selection tool.

② Pick up a cell in the image and draw a contour enclosing its red autofluorescence regions. Then, randomly select a region outside the cell as the background.

③ Use the measure function in ImageJ to calculate the mean fluorescence intensity of each selected area.

④ Subtract the background intensity from the fluorescence intensity of each area to obtain the autofluorescence intensity of each cell. At least 30 cells per experimental group should be selected for statistical analysis.

To acquire the two-photon red autofluorescence spectra from DNA oligonucleotides, we followed a specific protocol.

a. First, we used the two-photon microscope imaging system to locate the glass-water interface by detecting its third-harmonic generation (THG).

b. An aqueous solution containing the DNA oligonucleotides was then added onto a glass slide.

c. The microscope stage Z-axis was raised by 20 µm to move the focal plane into the solution.

d. A 1060 nm two-photon laser was used for excitation, with the following parameters: zoom 5, 10 mW laser power, 50-70% laser output power, and HV 220.

e. Spectral data was initially acquired with a cycle time of 10 s.

f. If a characteristic emission peak at 600 nm was not detected, the cycle time was adjusted to 1 second. The "live" function was then used to acquire real-time spectral data while manually adjusting the Z-axis of the microscope stage.

g. Once the 600 nm emission peak became visible on the screen, the "live" mode was stopped, the cycle time was reset to 10 s, and the final emission spectrum was acquired for analysis.

2. Analysis of two-photon red autofluorescence lifetime in a cell

Excited at 1060 nm, we acquired the two-photon fluorescence lifetime imaging microscopy of Ag-DNA autofluorescence. The collection wavelength range is 500-650 nm. To avoid photodamage on cells, each image was acquired within 120 s, and the laser power after the objective (40×, NA = 1.15) is 10 mW.

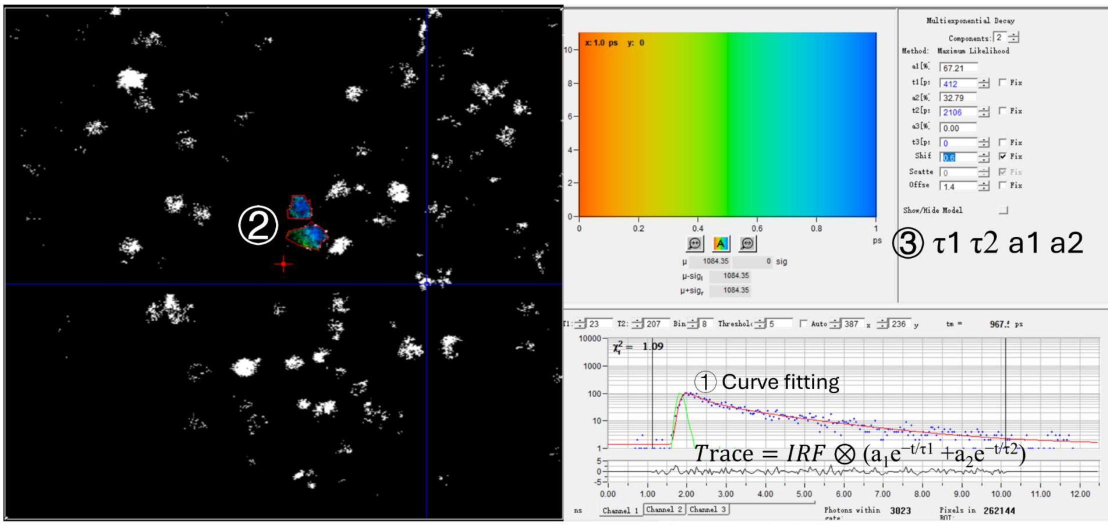

Supplementary Figure 11. The lifetime image processing interface of SPCImage software. The image processing steps:

① In the SPCImage software (Becker & Hickl GmbH), load the instrument response function (IRF, green curve) to convolve two-component exponential decay model, resulting in a fitting function $f(t) = IRF \otimes (a_1 e^{-t/\tau 1} + a_2 e^{-t/\tau 2})$. By varying parameters of shift, offset, τ1, τ2, a1 and a2, minimize the reduced chi-square value χ_r^2 to 1 as close as possible. Chi-square value is the index for good fitting between fitting curve (red curve) and the fluorescence decay statistics (blue dots).

② Use the "Define mask" tool in SPCimage software to enclose the red autofluorescence regions of interest (ROI) in cells on the lifetime image.

③ Extract the τ1, τ2, a1 and a2 parameters in each cell and gather red autofluorescence lifetime data from at least 30 cells.

3. Evaluating colocalization between lysosomes and Ag-mtDNA

For colocalization analysis, ImageJ's coloc plugin was employed to quantify the degree of spatal overlap between lysotracker's (green color) and Ag-DNA's (red color) fluorescence signals in Figure 1D and Figure 4A. The analysis included the calculation of Pearson's correlation coefficient and Manders' overlap coefficients, which are commonly used metrics for assessing colocalization. Pearson's correlation coefficient (PCC) evaluates the linear relationship between the intensities of two channels, with values ranging from -1 to +1, where +1 indicates perfect positive correlation, 0 represents no correlation, and -1 suggests complete negative correlation. This metric is independent of signal intensity and is widely used to assess the strength of colocalization between the images of two fluorophores. On the other hand, Manders' overlap coefficients (M1 and M2) measure the fraction of each signal that overlaps with the other. M1 represents the proportion of the first channel image that overlaps with the second channel one, while M2 reflects the proportion of the second channel image overlapping with the first channel one. These values range from 0 to 1, with higher values indicating greater overlap. The threshold for colocalization was set using Costes' randomization method, which generates randomized images to calculate a significance threshold, reducing bias from background noise. Colocalization is considered significant when PCC values exceed 0.5, or when Manders' coefficients are greater than 0.7, indicating a substantial degree of overlap between two image signals.

In the colocalization analysis using ImageJ's coloc function, the results include several key metrics: Pearson's R value (below threshold), Manders' M1 (Above zero intensity of Ch2), and Manders' tM1 (Above autothreshold of Ch2). The threshold refers to a user-defined or automated intensity cutoff applied to each channel, typically to exclude background noise or non-specific signal from the analysis. The Pearson's R value calculated below this threshold assesses the correlation between the two channels in the regions where the intensity values fall below the set threshold, helping to separate meaningful signal from noise. Zero intensity indicates that the Manders' M1 coefficient is calculated based on pixels in Ch2 where the intensity is greater than zero, meaning only the regions where signal is present are considered. This helps focus the analysis on the overlapping signal rather than areas with no fluorescence. Autothreshold refers to an automatically determined intensity threshold, calculated by the software based on the image's intensity distribution. In this case,

Manders' tM1 is calculated based on the pixels in Ch2 that exceed this autothreshold, allowing for a more unbiased analysis by eliminating the need for manual thresholding and ensuring the comparison is focused on relevant signal regions. The table below lists the Pearson's coefficients and Manders' coefficients of colocalization analysis in different types of cell death:

	Apoptosis	Ferroptosis	Pyroptosis	Necroptosis	Mitophagy
Pearson's R value (no threshold)	0.97	0.86	0.93	0.77	0.85
Pearson's R value (below threshold)	-0.04	-0.02	-0.01	-0.01	-0.03
Pearson's R value (above threshold)	0.96	0.83	0.92	0.62	0.81
Manders' M1 (Above zero intensity of Ch2)	0.991	0.996	0.992	0.915	0.993
Manders' M2 (Above zero intensity of Ch1)	0.995	0.995	0.993	0.698	0.991
Manders' tM1 (Above autothreshold of Ch2)	0.981	0.964	0.977	0.828	0.922
Manders' tM2 (Above autothreshold of Ch1)	0.982	0.964	0.979	0.663	0.925

4. Analysis of cell death by Annexin V/PI flow cytometry assay

In an Annexin V/PI flow cytometry assay, cells are stained with Annexin V, which binds to phosphatidylserine on the outer leaflet of the plasma membrane, and propidium iodide (PI), which stains DNA in cells with compromised membranes. The results are typically displayed in a scatter plot partitioned with four quadrants:

(1) Upper Left Q1 quadrant (Annexin V-/PI+): This quadrant represents necrotic cells that are negative for Annexin V but positive for PI. These cells have compromised membranes but have not externalized phosphatidylserine.

(2) Upper Right Q2 quadrant (Annexin V+/PI+): This quadrant represents late apoptotic or necrotic cells that are positive for both Annexin V and PI. These cells have lost membrane integrity and are in the later stages of apoptosis or have undergone necrosis.

(3) Lower Right Q3 quadrant (Annexin V+/PI-): This quadrant represents early apoptotic cells that are positive for Annexin V but negative for PI. These cells have externalized phosphatidylserine but still have intact membranes.

(4) Lower Left Q4 quadrant (Annexin V-/PI-): This quadrant represents live cells that are negative for both Annexin V and PI staining. These cells have intact membranes and are not undergoing apoptosis or necrosis.

In flow cytometry, cells undergoing ferroptosis, necroptosis, and pyroptosis have data points in Q2 of annexin V and PI stained cytometry (Figure S4), which is used to determine cell death rates.

5. Determination of lysosomal pH values in cell death

To measure lysosomal pH in different types of cell death, a pHLys Red probe (Dojindo) was used. Exciting at 561 nm, the red fluorescnece of probes passed the emission filter (pass band = 570-620 nm) and was detected by photomultiplier tubes in the confocal microscopy system. The fluorescence intensity of pHLys Red showed sensitive changes within the lysosomal pH range of 4.0-5.5. Reference solutions with pH values of 4.0, 4.5, 5.0, 5.5, 6.0, 6.5, and 7.0 were prepared, and their red fluorescence images (Figure S12) were acquired by a confocal fluorescnece microscope (Nikon Ti2-E A1R). Fluorescence intensity was calculated using ImageJ for each pH, and the corresponding data were used to generate a standard curve. Using the same method, fluorescence intensity in different cell death models was measured after applying the probe. The mean pH values and confidence intervals were calculated based on the measured fluorescence intensities and the standard curve. To calculate the confidence intervals, the mean intensity (u) of the fluorescence signal was first determined, followed by the calculation of the sample standard deviation (s). The standard error of the mean (SEM) was then calculated as $SEM = \frac{s}{\sqrt{n}}$, where n represents the sample size (n = 10). The 95% confidence

interval for the fluorescence intensity was subsequently computed as (u − 1.96 × SEM, u + 1.96 × SEM). The corresponding pH values of upper and lower intensity bound on the calibration curve is the 95% confidence interval for the measured pH.

	Fluorescence Intensity 95% CI	pH value 95% CI
Control	25.58-37.53	4.38-4.58
Apoptosis	41.27-54.8	4.18-4.33
Ferroptosis	19.63-30.88	4.48-4.74
Pyroptosis	24.71-38.88	4.36-4.60
Necroptosis	18.11-25.6	4.58-4.80
Mitophagy	39.46-50.42	4.22-4.35

To construct the standard curve and determine the corresponding pH values from fluorescence intensities obtained in various cell death models, a custom Python script was implemented using several essential libraries, including NumPy, Matplotlib, and SciPy. The script first defines the experimental data points, where the pH values and corresponding fluorescence intensities were stored as arrays using the NumPy package.

For data smoothing and curve fitting, SciPy's make_interp_spline function was used to perform cubic spline interpolation, generating a smooth curve between the given pH values (x-axis) and fluorescence intensities (y-axis) over the range of pH 4 to 7. A finer set of x-values was created using NumPy's linspace function, and the corresponding y-values were interpolated using the cubic spline.

To visualize the data, Matplotlib was utilized to plot both the original experimental data points (as red scatter points in the figure below) and the interpolated smooth curve (as a blue line in the figure below), with the x-axis representing pH values and the y-axis representing fluorescence intensities. Grid lines and legends were added to enhance the clarity of the plot.

In addition to generating the standard curve, the script included a function to calculate the pH value corresponding to a given fluorescence intensity. This was done using SciPy's fsolve function, which solves for the x-value (pH) that satisfies the equation $y - spl(x) = 0$, where $spl(x)$ represents the cubic spline function. The function takes an initial guess (set to 5 in this case) and iteratively solves for the pH that corresponds to a specific fluorescence intensity.

The target fluorescence intensities measured from the cell death models were input into the script, and their corresponding pH values were calculated using the defined interpolation method and equation solver.

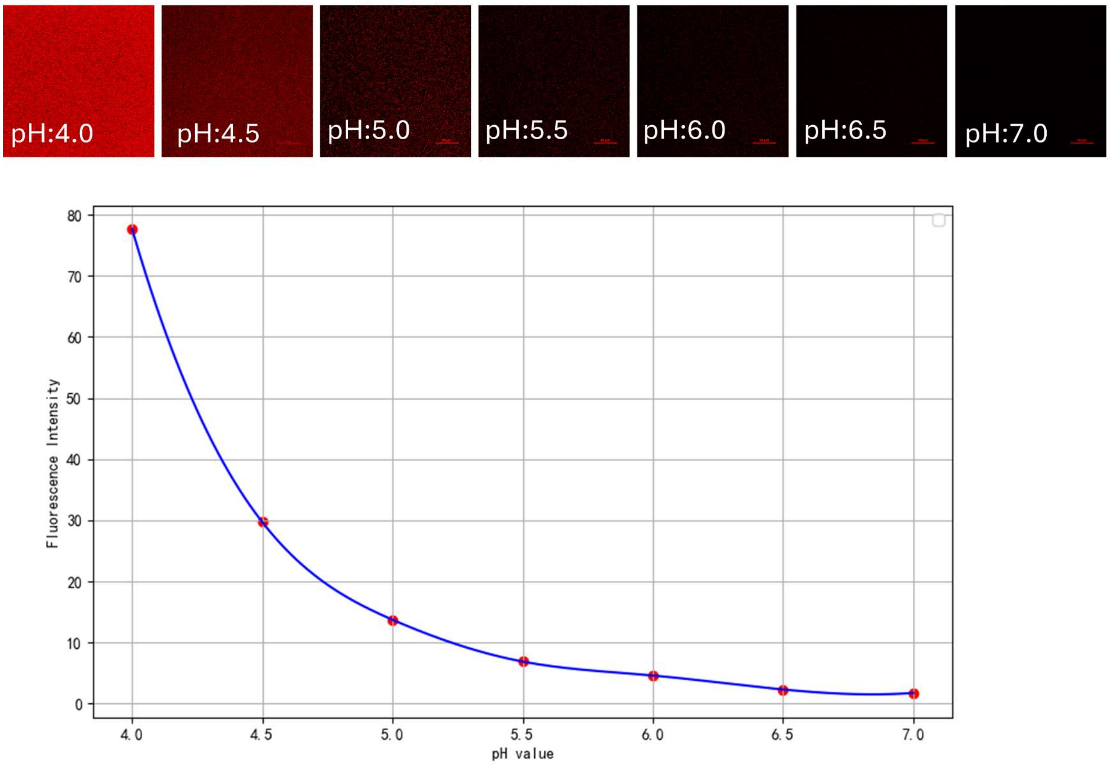

Supplementary Figure 12. Fluorescence images and corresponding standard curve of pHLys Red in reference solutions with pH values of 4.0, 4.5, 5.0, 5.5, 6.0, 6.5, and 7.0. Scale bars: 10 μm.

Based on the standard curve, we found that the lysosomal pH values in all groups ranged between 4 and 5 (Figure S6B), which is within the pH range required for dsDNA denaturation.

Below is the complete code used for generating the standard curve and calculating the corresponding pH values:

```
import numpy as np
import matplotlib.pyplot as plt
from scipy.interpolate import make_interp_spline, BSpline
from scipy.optimize import fsolve

# Set the font to SimHei, which supports Chinese characters
plt.rcParams['font.sans-serif'] = ['SimHei']
plt.rcParams['axes.unicode_minus'] = False
# Given data points
x_values = np.array([4, 4.5, 5, 5.5, 6, 6.5, 7])
y_values = np.array([77.676, 29.7, 13.708, 6.854, 4.569, 2.285, 1.714])
# Generate smooth curve
x_smooth = np.linspace(4, 7, 300)
spl = make_interp_spline(x_values, y_values, k=3)  # k=3 means cubic spline interpolation
```

```python
y_smooth = spl(x_smooth)

# Plot original data points and smooth curve
plt.figure(figsize=(10, 6))
plt.scatter(x_values, y_values, color='red')
plt.plot(x_smooth, y_smooth, color='blue')
plt.xlabel('pH value')
plt.ylabel('Fluorescence Intensity')
plt.legend()
plt.grid(True)
plt.show()

# Define a function to find the x value for a given y value
def find_x_for_y(y_target):
    # Define the equation y - spl(x) = 0
    equation = lambda x: spl(x) - y_target
    # Solve for the x value
    x_solution = fsolve(equation, 5)  # 5 is the initial guess
    return x_solution

# Given y values
y_targets = [48.65, 45.94, 23.53, 33.41, 20.91]
# Calculate corresponding x values
x_solutions = [find_x_for_y(y) for y in y_targets]

# Print the results
print("Corresponding x values for the given y values:", x_solutions)
```

6. Analysis of next generation sequencing

In this study, DNA fragments extracted from lysosomes were subjected to next-generation sequencing (NGS). The obtained sequencing reads were aligned to the reference genome using the BWA (Burrows-Wheeler Aligner) tool, which is designed for high-throughput sequence alignment. The alignment process involved the following steps: first, the raw sequencing data in FASTQ format were preprocessed to remove low-quality reads and adapter sequences. Then, the cleaned reads were mapped to the reference genome (human genome GRCh38) using BWA, which generates a SAM (Sequence Alignment/Map) file containing the alignment information.

Subsequently, the SAM file was converted into a BAM (Binary Alignment/Map) file using SAMtools, a suite of programs for manipulating alignments in the SAM format. SAMtools was used to sort the BAM file and index it, allowing for efficient access to alignment data. Following this, coverage and depth of sequencing were calculated for each chromosome using the SOAP.coverage software. This software computes the coverage metrics, including the total number of reads covering each base position, thereby providing insights into the uniformity of sequencing across the genome.

The analysis revealed that over 90% of the genomic regions could be covered by the sequencing reads, indicating a high level of sequencing depth and breadth. This comprehensive coverage is crucial for accurately assessing the genetic landscape of the lysosomal DNA fragments and further characterizing their role in cellular processes.

7. Photophysical Characterization of Aggregated DNA

The photophysical properties of aggregated DNA were systematically characterized using aggregated polyA (A21) oligonucleotides as a model system. The fluorescence quantum yield (Φ) was determined by a relative method using Rhodamine B in water as the standard (Φ_ref = 0.31). The photostability was assessed by continuously irradiating a 100 µM polyA sample with a 1060 nm two-photon laser and recording the fluorescence intensity at various time points up to 9 min. Spectroscopic analysis revealed a broad absorption spectrum spanning the visible range from 460 nm to 590 nm. Consistent with this, the fluorescence excitation spectrum, obtained by monitoring the emission at 620 nm, confirmed a broad excitation range primarily between 450 nm and 600 nm. Finally, the single-photon fluorescence emission spectrum was recorded upon excitation at 530 nm, showing the characteristic emission peak centered at approximately 600 nm.

Supplementary Table 1. Fluorescence Quantum Yield of Aggregated A21 Oligonucleotide.

	Absorbance (A)	Integrated Intensity (I)	Quantum Yield (Φ)
AIE-DNA	0.030	3,352,655	0.0014
Rhodamine B	0.038	940,300,530	0.31

Footnote. The fluorescence quantum yield (Φ_{DNA}) was determined by the relative method using the formula: $\Phi_{DNA} = \Phi_{RB} \times (I_{DNA} / I_{RB}) \times (A_{RB} / A_{DNA})$ [58]. Rhodamine B in water was used as a reference standard (Φ_{RB} = 0.31, literature value) [59]. All samples were excited at 530 nm, and the emission spectra were integrated over the wavelength range of 565–700 nm to obtain the integrated intensities (I). The absorbance (A) at 530 nm is listed for each sample. The concentrations used for this measurement were 1 mM for AIE-DNA and 0.45 μM for Rhodamine B.

8. Preparation of Oligonucleotide Solutions

Custom-synthesized DNA oligonucleotides were dissolved in nuclease-free ddH_2O to prepare a stock solution with a final concentration of 100 μM. To ensure accurate and reproducible spectroscopic measurements, a minimum sample volume of 40 μL was maintained for all subsequent experiments.

Supplementary Table 2. Design of DNA oligos with various sequences

Oligonucleotide	Sequence (5' - 3')
NG16	GGGTGGGTTGGGTGGG
EAD	CTGGGTGGGTGGGTGGGA
TT3T	GGGTGGGTTTGGGTGGG
TBA	GGTTGGTGTGGTTGG
Hum21	GGGTTAGGGTTAGGGTTAGGG
h-Telo	AGGGTTAGGGTTAGGGTTAGGGT
HT	TTGGGTTAGGGTTAGGGTTAGGGA
PW17	GGGTAGGGCGGGTTGGG
Ds26	CAATCGGATCGAATTCGATCCGATTG
Ds17-1	CCAGTTCGTAGTAACCC
Ds17-2	GGGTTACTACGAACTGG
A21	AAAAAAAAAAAAAAAAAAAAA
T21	TTTTTTTTTTTTTTTTTTTTT
C21	CCCCCCCCCCCCCCCCCCCCC
G21	GGGGGGGGGGGGGGGGGGGGG

Supplementary Table 3. The longest consecutive match rate of the oligos to mtDNA

Oligonucleotide	Longest Consecutive Match	Match Rate
NG16	9	56.25%
EAD	10	55.56%
TT3T	11	64.71%
TBA	11	73.33%
Hum21	11	52.38%
h-Telo	11	47.83%
HT	11	45.83%
PW17	9	52.94%
Ds26	8	30.77%
Ds17-1	10	58.82%
Ds17-2	10	58.82%
A21	8	38.10%
T21	8	38.10%
C21	9	42.86%
G21	9	42.86%

Supplementary Table 4. D-loop sequence of human mtDNA

Oligonucleotide	Sequence (5' - 3')
D-loop 1	ATAACAAAAAATTTCCACCAAA
D-loop 2	AACAAACCTACCCACCCTTAA
D-loop 3	AAAATAATGTGTTAGTT

9. Clinical study

The current study employed a case-control design involving a healthy control group (n = 22) and a sepsis group of patients (n = 14), recruited from Zhujiang Hospital, Guangzhou, China, between 2023 and 2024. Venous blood samples (10 mL) were collected from all participants to measure Procalcitonin (PCT) levels and blood autofluorescence intensity. Samples from sepsis patients were collected within 24 h of hospitalization. Sepsis diagnosis followed the Sepsis-3 criteria (Singer et al., 2016), defined by a SOFA or qSOFA score of ⩾ 2 points, confirmed by a physician. Healthy controls were physically healthy individuals with no underlying diseases and no hospitalization history within the past two years. Exclusion criteria included minors, pregnant individuals, those with mental illnesses, and other vulnerable populations. Ethical approval was granted by the Institutional Review Board of Zhujiang Hospital (Approval No. 2022-KY133-01), and all participants provided written informed consent. Detailed demographic and clinical data of sepsis patients are presented in **Supplementary Table 5**.

Supplementary Table 5. Clinical characteristics of sepsis patients recruited in this study

	**Participant**	**Gender**	**Age (years)**	**SOFA score**	**PCT level (ng/mL)**	**Bacteria Species in Blood Culture**
			**Positive**			
C3-1	No. 1	Male	66	8	0.15	*Enterococcus faecalis*
C30-1	No. 2	Male	27	6	63.76	*Candida albicans*
C39-1	No. 3	Male	35	10	＞200	*Aspergillus, Staphylococcus aureus*
C54-1	No. 4	Male	42	8	2.79	*Hemolytic staphylococcus*
			**Negative**			
C10-1	No. 5	Male	49	8	0.05	-
C11-1	No. 6	Male	50	12	2.48	-
C1-1	No. 7	Male	70	9	3.20	-
C18-1	No. 8	Male	59	2	44.14	-
C19-1	No. 9	Male	76	8	11.41	-
C71-1	No. 10	Male	41	11	35.64	-
C9-1	No. 11	Female	73	9	0.13	-
C29-1	No. 12	Female	67	10	3.10	-
C69-1	No. 13	Female	73	10	52.92	-
C56-1	No. 14	Female	53	14	-	-

SOFA: sequential organ failure assessment; PCT: procalcitonin
